# Efficacy and Safety of Newly Diagnosed Multiple Myeloma Combination Therapies: A Systematic Review Integrating Network Meta-Analysis and Real-World Vigilance Study

**DOI:** 10.3390/ph19010018

**Published:** 2025-12-21

**Authors:** Yanjun Liu, Ying Zhang, Wenhui Yang, Haoyan Du, Shijie Sun, Zuojing Li, Dongsheng Zong

**Affiliations:** 1 School of Clinical Pharmacy, Shenyang Pharmaceutical University, Shenyang 110016, China; yjl3202@163.com; 2 National Medical Products Administration, Beijing 100037, China; zhangying@nmpa.gov.cn; 3 School of Medical Devices, Shenyang Pharmaceutical University, Shenyang 110016, China; qudaoshiji@163.com (W.Y.); 2519224662ddd@gmail.com (H.D.); sunshijie1222@163.com (S.S.)

**Keywords:** multiple myeloma, daratumumab, network meta-analysis, FAERS database, adverse drug events

## Abstract

**Background**: Although anti-CD38 monoclonal antibody-based regimens are standard care for newly diagnosed multiple myeloma (NDMM), direct comparative efficacy and comprehensive real-world safety data remain scarce. **Methods**: We conducted a systematic review and Bayesian network meta-analysis (NMA) of randomized controlled trials (RCTs). Efficacy was assessed using hazard ratios (HRs) for progression-free survival and odds ratios (ORs) for response rates, with treatment rankings evaluated by Surface Under the Cumulative Ranking (SUCRA) values. Separately, adverse event reports for daratumumab, bortezomib, lenalidomide, and dexamethasone (D_VRd) regimens were extracted from the US FDA Adverse Event Reporting System (FAERS) (Q1 2015–Q2 2025). Statistical analyses were performed using R (4.3.3) and STATA (16.0). **Results**: The NMA included 33 RCTs. For the primary efficacy endpoints, compared to the standard bortezomib, lenalidomide, and dexamethasone (VRd) regimen, both D_VRd (OR = 3.21, 95% CI: 2.46–4.26; HR = 0.48, 95% CI: 0.38–0.63) and isatuximab plus VRd (Isa_VRd) (OR = 1.71, 95% CI: 1.25–2.32; HR = 0.66, 95% CI: 0.51–0.85) regimens demonstrated superior efficacy. Subsequent pharmacovigilance analysis of D_VRd identified 11,714 FAERS reports, yielding 197 significant adverse drug event signals (64 unlabeled). These signals primarily affected elderly males and showed a bimodal distribution pattern. **Conclusions**: Combination regimens containing anti-CD38 monoclonal antibodies demonstrate superiority in achieving deep remission and survival benefits, with D_VRd and Isa_VRd regimens showing particularly outstanding performance. However, efficacy and safety profiles vary across different combination regimens. Real-world data analysis further indicates that the D_VRd regimen carries several safety risk signals that remain underappreciated and exhibits a bimodal time distribution pattern. These findings provide new evidence to guide clinical decision-making and risk-stratified monitoring.

## 1. Introduction

Multiple myeloma (MM) is a malignant clonal disease originating from bone marrow plasma cells [1]. It accounts for approximately 10% of hematologic malignancies and 1.2% of all cancer-related deaths [2]. The disease typically evolves from monoclonal gammopathy of undetermined significance (MGUS) [3]. Its incidence continues to rise alongside an aging population, with a median diagnostic age of 66 years and a significantly higher risk observed in males [4,5]. Despite significant improvements in patient survival achieved through proteasome inhibitors (PIs), immunomodulatory drugs (IMiDs), and autologous stem cell transplantation (ASCT) [6], MM remains incurable. The vast majority of patients ultimately progress to relapsed/refractory multiple myeloma (RRMM). As the line of therapy advances, therapeutic options become increasingly limited, and efficacy diminishes [7], constituting a major challenge in clinical management.

The therapeutic landscape for induction therapy in NDMM has continuously evolved. Since triple-agent regimens were established as a standard in 2015, the VRd regimen has become mainstream, competing with carfilzomib-based triple regimens (KRd) based on patient risk stratification [8,9,10,11]. Since 2022, the emergence of quadruplet regimens has challenged the dominance of triple regimens. Several pivotal phase III clinical trials (e.g., GMMG-HD7, PERSEUS, CASSIOPEIA, and IsKia) have demonstrated [12,13,14,15] that quadruplet regimens containing anti-CD38 monoclonal antibodies offer superior progression-free survival (PFS) and depth of response compared to traditional regimens, leading to their incorporation into first-line treatment recommendations by European and American guidelines [16,17]. However, disparities exist in the recommendation levels for specific combination regimens across different national guidelines, and classic triple-agent therapies retain significant value in resource-limited settings. While multiple RCTs provide high-level evidence for the efficacy of individual monoclonal antibody regimens, the lack of head-to-head comparative evidence between these regimens makes it challenging for clinicians to directly quantify the relative advantages in efficacy and safety. Furthermore, RCTs are constrained by sample size, follow-up duration, and highly selective patient enrollment, often failing to comprehensively capture all potential adverse events—particularly rare, delayed, or subgroup-specific occurrences.

Therefore, integrating high-level indirect comparative evidence with large-scale real-world safety data is crucial to address this evidence gap. NMA enables a comprehensive assessment of the relative efficacy and safety of different treatment options by integrating direct and indirect comparative evidence across multiple interventions [18]. Concurrently, FAERS, as the world’s largest post-marketing drug safety surveillance database, aggregates extensive real-world spontaneous reporting data, serving as a vital tool for comprehensively evaluating post-marketing drug safety [19].

Based on this rationale, the present study integrates NMA with real-world pharmacovigilance methods. It aims to systematically compare the efficacy and safety profiles of various induction regimens for NDMM, including conducting subgroup analyses for transplant-eligible (TE) and transplant-ineligible (TIE) patients. Furthermore, by leveraging the FAERS database, this study performs an in-depth data-mining analysis of adverse drug events associated with the D_VRd to delineate its safety profile and identify potential risk signals not documented in the prescribing information.

## 2. Results

### 2.1. Results of Network Meta-Analysis

#### 2.1.1. Study Overview and Patient Characteristics

**Figure 1:**
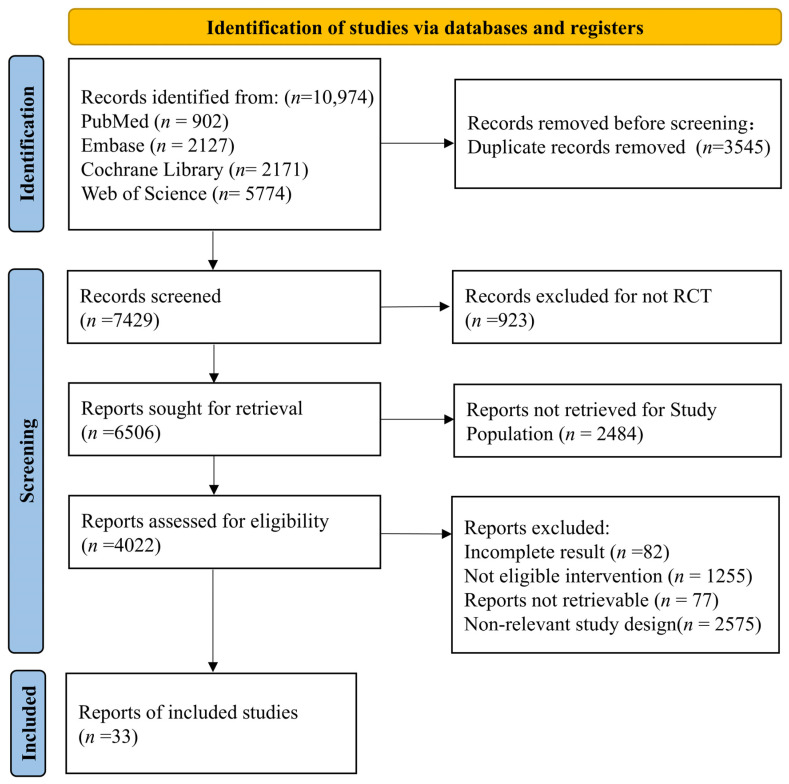
Literature Screening Flowchart.

A total of 33 RCTs [12,13,14,15,20,21,22,23,24,25,26,27,28,29,30,31,32,33,34,35,36,37,38,39,40,41,42,43,44,45,46,47,48] were ultimately included, involving 12,782 patients(Figure 1). Among these, 3825 patients received 11 monoclonal antibody-containing combination regimens (e.g., Isa_VRd, D_VRd), while 6534 patients received 8 standard triple regimens (e.g., VRd) (Table 1). The majority of the included studies were assessed as having a low overall risk of bias, with the main concern being a lack of blinding of participants and personnel (Supplementary Figure S1). The difference in Deviance Information Criterion (DIC) between the consistency and inconsistency models was less than 5 (Supplementary Table S1), indicating comparable model fit and no significant global inconsistency, thus supporting the reliability of the results.

#### 2.1.2. Primary Efficacy Outcomes

**Figure 2:**
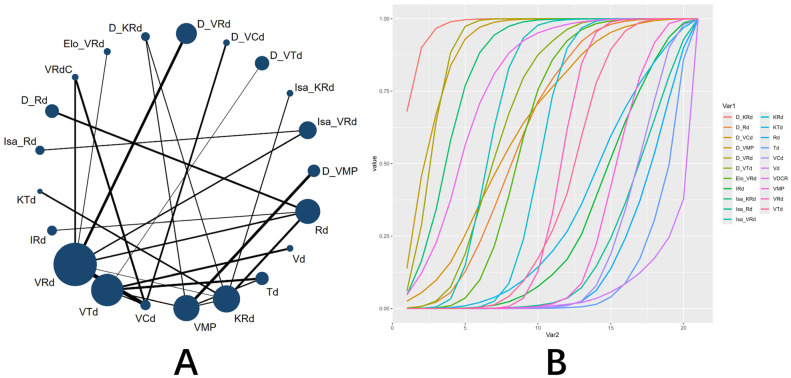
NMA of MRD negativity rate. (**A**) Network plot of treatment comparisons. The size of each node is proportional to the number of participants assigned to that regimen, and the width of each line (edge) is proportional to the number of trials directly comparing the connected regimens. (**B**) SUCRA plot. A higher SUCRA value (closer to 100%) indicates a greater probability of being the most effective regimen for improving CR. Abbreviations: D, daratumumab; Isa, isatuximab; Elo, elotuzumab; V, bortezomib; M, melphalan; P, prednisone; K, carfilzomib; R, lenalidomide; d, dexamethasone; T, thalidomide; C, cyclophosphamide; I, ixazomib.

**Figure 3:**
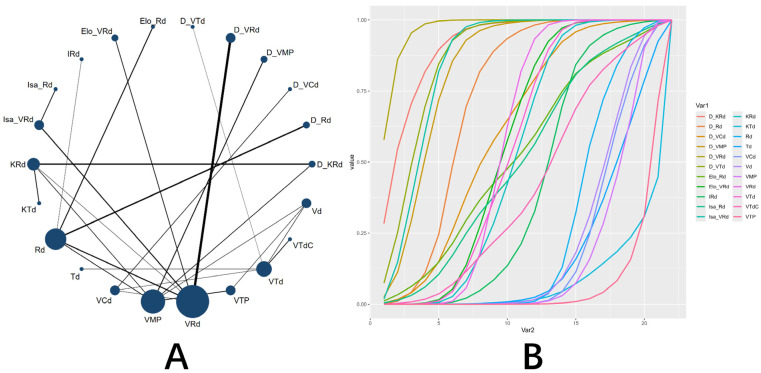
NMA of PFS. (**A**) Network plot of treatment comparisons. The size of each node is proportional to the number of participants assigned to that regimen, and the width of each line (edge) is proportional to the number of trials directly comparing the connected regimens. (**B**) SUCRA plot. A higher SUCRA value (closer to 100%) indicates a greater probability of being the most effective regimen for improving OS. Abbreviations: D, daratumumab; Isa, isatuximab; Elo, elotuzumab; V, bortezomib; M, melphalan; P, prednisone; K, carfilzomib; R, lenalidomide; d, dexamethasone; T, thalidomide; C, cyclophosphamide; I, ixazomib.

For the key efficacy endpoints, regimens containing anti-CD38 monoclonal antibodies demonstrated significant advantages over the standard VRd regimen. Regarding the MRD negativity rate, the SUCRA indicated daratumumab, carfilzomib, lenalidomide, and dexamethasone (D_KRd) as optimal (0.977), followed by D_VRd (0.894) (Figure 2). Compared with VRd, D_KRd (OR = 4.83, 95% CI: 2.67–8.88), D_VRd (OR = 3.21, 95% CI: 2.46–4.26), isatuximab carfilzomib, lenalidomide, and dexamethasone (Isa_KRd) (OR = 2.67, 95% CI: 1.38–5.27), and Isa_VRd (OR = 1.71, 95% CI: 1.25–2.32) all achieved significantly higher MRD negativity rates ( Supplementary Figure S2). In terms of PFS, D_KRd (HR = 0.55, 95% CI: 0.33–0.93), D_VRd (HR = 0.48, 95% CI: 0.38–0.63), daratumumab, bortezomide, thalidomide, and dexamethasone (D_VTd) (HR = 0.63, 95% CI: 0.42–0.94), and Isa_VRd (HR = 0.66, 95% CI: 0.51–0.85) showed superior and statistically significant performance compared to VRd(Supplementary Figure S3). SUCRA further confirmed the superiority of daratumumab-containing regimens in prolonging PFS. The top 7 regimens were D_VRd (0.970), D_KRd (0.909), D_VTd (0.870), Isa_VRd (0.850), daratumumab, bortezomide, melphalan, and prednisone (D_VMP) (0.826), daratumumab, lenalidomide, and dexamethasone (D_Rd) (0.722), and daratumumab, bortezomide, cyclophosphamide, and dexamethasone (D_VCd) (0.614) (Figure 3).

#### 2.1.3. Other Efficacy Outcomes and Subgroup Analyses

Regarding ORR, compared with the VRd regimen, D_VRd (OR = 2.78, 95% CI: 1.5–5.37), Isa_KRd (OR = 3.44, 95% CI: 1.02–14.14), and Isa_VRd (OR = 1.6, 95% CI: 1–2.6) demonstrated statistically significant superior efficacy. Furthermore, an improvement in ORR was also observed for D_KRd (OR = 2.38, 95% CI: 0.75–9.48) and D_VTd (OR = 2.14, 95% CI: 0.78–5.9), although these differences did not reach statistical significance (Supplementary Figures S4 and S5). Regarding the CR, the Isa_VRd regimen demonstrated the most prominent performance. Among daratumumab-based regimens, D_VRd (0.825) and D_VTd (0.813) ranked highest (Supplementary Figures S6 and S7). In the current analysis for OS, the D_VTd regimen ranked first. However, while most regimens showed a trend toward OS benefit, none reached statistical significance compared to VRd (Supplementary Figures S8 and S9), indicating that longer-term follow-up data are required to confirm their definitive impact on OS.

**Figure 4:**
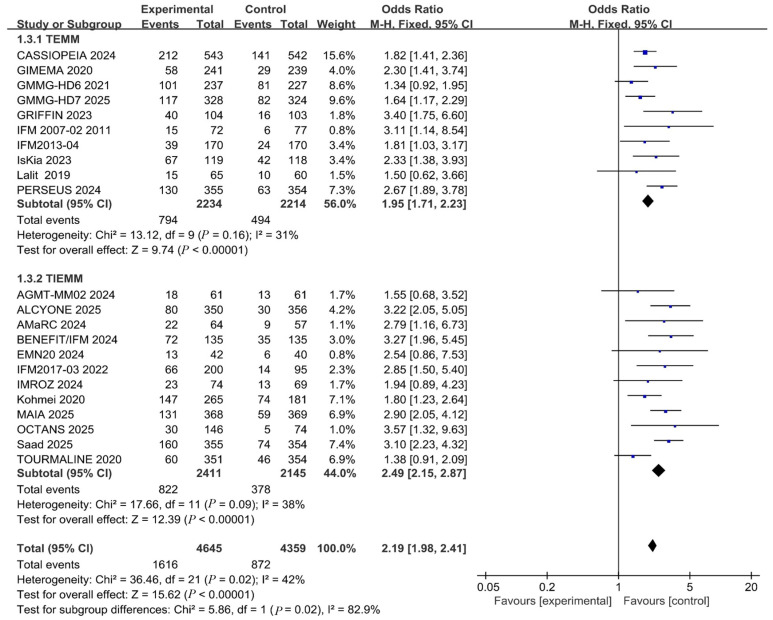
Subgroup analysis of TE and TIE populations for the MRD negativity rates outcome.

Subgroup analyses based on transplant eligibility demonstrated consistent benefits of anti-CD38 monoclonal antibodies across TE and TIE subgroups for outcomes including ORR, CR, PFS, and OS, with no statistically significant differences between groups (Supplementary Figures S10–S13). However, regarding the MRD negativity rate, the OR in the TIE subgroup (OR = 2.49 [2.15, 2.87], I^2^ = 38%) was significantly higher than that in the TE subgroup (OR = 1.87 [1.64, 2.14], I^2^ = 31%). With a statistically significant *p* = 0.02 (<0.05) for subgroup comparison (Figure 4). This suggests that while anti-CD38 monoclonal antibodies provide universal benefit across patient populations, they may be particularly advantageous in helping TIE patients achieve deeper molecular responses.

#### 2.1.4. Safety Outcomes

The safety analysis revealed that adding an anti-CD38 monoclonal antibody to standard regimens generally increased the risk of grade ≥ 3 AEs and the likelihood of treatment discontinuation. SUCRA indicated that the regimens with the highest risk were D_KRd (0.086) and Isa_KRd (0.109), suggesting that combining an anti-CD38 monoclonal antibody with carfilzomib-based regimens further exacerbates toxicity (Supplementary Figures S14–S17). With respect to hematologic toxicity and infection risk, D_KRd (0.135; 0.222) and Isa_KRd (0.103; 0.343) were similarly ranked highest for the respective risks (Supplementary Figures S18–S21). In contrast, the isatuximab, lenalidomide, and dexamethasone (Isa_Rd) regimen (0.943) was associated with the lowest risk of infections. Analysis of neurotoxicity indicated that adding anti-CD38 monoclonal antibodies to bortezomib-containing triple regimens did not significantly alter neurotoxicity risk levels. The regimens with the highest neurotoxicity remained the traditional VTd (0.034) and VMP (0.187) regimens, consistent with the known toxicity profile of bortezomib (Supplementary Figures S22 and S23).

### 2.2. Real-World Pharmacovigilance Study Results

#### 2.2.1. Report Characteristics and System Organ Class (SOC) Distribution

Following the removal of duplicate reports, a total of 19,026,509 reports were screened. Among these, 11,714 reports were associated with the D_VRd regimen, involving 3861 distinct patients (Supplementary Figure S24). Physicians submitted the majority of reports (51.5%). Patients were predominantly male (45.5%) and elderly, aged 65–85 years (38.8%). The most frequently reported serious outcome was prolonged hospitalization (28.1%) (Supplementary Table S2). Adverse reactions primarily involved the following SOCs: General disorders and administration site conditions (*n* = 1714, 17.3%), Infections and infestations (*n* = 1624, 16.39%), Neoplasms benign, malignant and unspecified (incl cysts and polyps) (*n* = 1120, 11.3%), Blood and lymphatic system disorders (*n* = 986, 9.95%), and various Nervous system disorders (*n* = 835, 8.43%) (Supplementary Figure S25).

#### 2.2.2. High-Frequency and Strong-Signal AEs

Among the adverse events reported for the D_VRd regimen, the most frequently reported included peripheral neuropathy and neutropenia, among others. Notably, infectious pneumonia, fatigue, diarrhea, rash, decreased white blood cell count, and urinary tract infection did not meet the signal detection threshold, suggesting a weaker association with the treatment regimen (Table 2).Using the Reporting Odds Ratio (ROR) and the Bayesian Confidence Propagation Neural Network (BCPNN) methods for signal detection, with strength categorized based on the IC025 value, the analysis identified a total of 197 significant safety signals. Among these, 25 signals were classified as strong (IC025 > 3) and 95 as moderate (1.5 < IC025 ≤ 3). The strong signals are predominantly concentrated in severe and varied infections and their complications, such as pneumomediastinum, pulmonary mucormycosis, BK virus infection, and hemorrhagic cystitis (Supplementary Table S3). The distribution pattern indicates that infection-related risks may represent a core safety issue requiring prioritized monitoring and management in the clinical application of the D_VRd regimen.

#### 2.2.3. Temporal Distribution and Label Comparison

**Figure 5:**
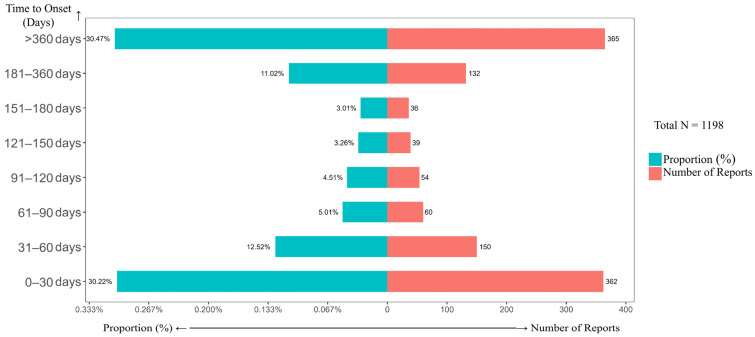
Time-to-onset distribution of adverse events for the D_VRd regimen.

Analysis of the time-to-onset of adverse events demonstrated a typical bimodal distribution pattern (Figure 5). The first peak occurred during the early treatment phase (0–30 days, 30.22%), likely attributable to the direct toxic effects of the drugs. A second peak emerged in the mid-to-late phase of treatment (>360 days, 30.47%), suggesting that this period may be dominated by cumulative and delayed toxicities. Through systematic comparison with the product label, this study for the first time identified 64 novel safety signals not previously listed (Table 3), providing novel critical targets for clinical monitoring.

For each time interval shown on the Y-axis, the associated proportion (%) (left X-axis) and number of reports (right X-axis) are presented. The analysis included all adverse event reports for the D_VRd regimen that contained complete time-to-onset information.

## 3. Discussion

The study systematically evaluated the efficacy and safety of multiple induction regimens for NDMM by integrating NMA with large-scale real-world pharmacovigilance data. Results confirmed that quintuple regimens containing anti-CD38 monoclonal antibodies—D_VRd, D_VTd, D_KRd, Isa_VRd, and Isa_KRd—ranked among the top five in overall efficacy, strongly supporting their prioritization as first-line treatments for NDMM. This outcome reflects a shift in current treatment philosophy from the traditional “stepwise addition” approach to a strategy focused on achieving the “maximal depth of response” [49]. Among the top-performing regimens, the top three daratumumab-based and the subsequent two isatuximab-based schemes exhibit distinct characteristics. Daratumumab exerts multiple effects, including complement-dependent cytotoxicity (CDC), antibody-dependent cellular cytotoxicity (ADCC), and direct induction of apoptosis [50,51], demonstrating broad synergistic potential with various backbone regimens. In contrast, isatuximab targets a specific epitope on CD38, exhibiting potent direct pro-apoptotic activity [52,53]. Its therapeutic advantage is fully realized only when combined with mechanism-matched regimens (VRd) or potent regimens (KRd), demonstrating unique potential for deep tumor clearance. Its potent direct pro-apoptotic mechanism likely plays a key role in this effect.

The central finding of this study is that, while quadruplet regimens are generally superior to triplet regimens, significant differences exist among various drug combinations in terms of efficacy (e.g., ORR, MRD, PFS) and toxicity. This heterogeneity fundamentally stems from their distinct synergistic mechanisms and additive toxic effects. Specifically, the D_VRd regimen demonstrated the most significant PFS benefit, attributable to the multifaceted mechanisms of daratumumab (ADCC, CDC) [54,55] producing extensive synergistic antitumor effects with VRd, as well as sustained drug exposure due to its prolonged half-life [56]. However, both NMA and real-world data indicate a high toxicity risk with this regimen, particularly regarding hematologic toxicity and infections. This may occur because daratumumab, while targeting tumor cells, may also affect CD38-expressing hematopoietic progenitor cells. Furthermore, the myelosuppressive properties of lenalidomide combined with bortezomib’s detrimental effects on the hematopoietic microenvironment synergistically exacerbate hematopoietic dysfunction. Concurrently, sustained drug exposure prolongs myelosuppression while maintaining efficacy, collectively contributing to hematologic adverse events such as neutropenia and thrombocytopenia. Additionally, through data mining of the FAERS database, this study for the first time systematically identifies several potential safety signals for the D_VRd regimen not currently listed in its prescribing information, primarily including severe opportunistic infections (such as pulmonary mucormycosis) and cardiotoxicity. The latter may be attributed to the synergistic effects of endothelial damage induced by lenalidomide, proteasome inhibition in cardiomyocytes by bortezomib [57], and immune dysregulation associated with daratumumab.

We observed that some adverse events with high reporting frequencies, such as infectious pneumonia and diarrhea, did not generate significant safety signals (i.e., ROR values close to or below 1, and negative IC025) (Table 2). The pattern highlights the core principle of signal detection in spontaneous reporting systems: it identifies relative differences in reporting rates (disproportionality) rather than absolute frequencies. Events like infectious pneumonia and diarrhea are commonly observed in patients with multiple myeloma, potentially due to the disease itself, concomitant therapies, or underlying conditions, resulting in a high baseline reporting rate in the FAERS database. Consequently, when the D_VRd regimen does not significantly increase the reporting rate of these events relative to this high background, the signal detection threshold is not met. This precisely illustrates that disproportionality analysis effectively screens for “abnormal” reports with a potentially stronger drug association, rather than merely listing all common adverse events. On the other hand, the D_KRd and Isa_KRd regimens demonstrate superior performance in MRD negativity rate and ORR, respectively. Their mechanisms likely involve the enhanced immune function of T cells and NK cells through the combined action of anti-CD38 monoclonal antibodies and the immunomodulator lenalidomide [51], coupled with a more potent synergistic pro-apoptotic effect when combined with carfilzomib, thereby achieving deeper disease remission. However, the direct inhibitory effect of carfilzomib on the bone marrow microenvironment [58], combined with the immune-activating state induced by monoclonal antibodies, results in a synergistic toxicity, leading to higher rates of grade ≥3 AEs and hematologic toxicity for both regimens. This underscores that clinical decision-making must shift from choosing the most potent regimen to selecting the best-matched regimen for the individual patient, necessitating a meticulous balance between achieving deep remission and maintaining treatment tolerability.

This study first revealed a “bimodal distribution” pattern in the timing of AEs associated with daratumumab combination therapy. The early peak (0–30 days) likely stems from acute direct drug toxicity, such as infusion-related reactions and bone marrow suppression, consistent with the known direct immune effects and cytokine release mechanisms of anti-CD38 monoclonal antibodies [59]. The mid-to-late peak (181–360 days) may be associated with cumulative toxicity and delayed infections caused by prolonged immunosuppression, a phenomenon also reported in long-term immunosuppressive therapies [60]. Given this temporal distribution pattern, we recommend incorporating treatment duration as a key parameter in patient risk assessment. Establishing targeted clinical monitoring windows for different treatment phases would enable early detection of potential adverse events and prompt intervention.

Subgroup analysis revealed a key finding: although anti-CD38 mAbs provided comparable PFS and OS benefits for both TE and TIE patients, significantly greater improvement in MRD negativity rates was observed in TIE patients (subgroup difference *p* = 0.02). This finding carries substantial clinical implications, suggesting that immunotherapy may more effectively activate anti-tumor immune responses and achieve deeper molecular remission in older or frail patients with age-related immune system alterations. This observation provides important insights for the emerging field of “geriatric immuno-oncology” and warrants further validation through prospective studies. Additionally, this study precisely positioned different regimens within the framework of a network meta-analysis. While the MAIA trial established the role of the D_Rd regimen, our analysis demonstrates that the Isa_Rd regimen offers both remarkable efficacy (ranking second in CR) and favorable safety (particularly manifesting as low infection risk). In our analysis, the Isa_Rd regimen demonstrated a high CR ranking alongside the most favorable safety profile in terms of infection risk, presenting a distinct efficacy-safety characteristic compared to other regimens.

In summary, this study integrates evidence on the efficacy and safety of induction regimens for NDMM, providing an evidence-based foundation for individualized treatment strategies to aid in optimizing clinical decision-making. Simultaneously, based on the identified potential safety signals and their possible mechanisms, clinicians should enhance preventive screening, early intervention for specific opportunistic infections (e.g., fungal, viral), and establish regular cardiac function monitoring protocols for patients receiving the D_VRd regimen. This approach aims to improve overall treatment efficacy and safety, thereby advancing clinical practice toward greater precision and personalization.

While this study provides valuable evidence-based guidance for selecting combination therapies in NDMM, several limitations must be acknowledged: (1) Variations in follow-up duration across the included trials may affect the stability of OS outcomes. (2) Considerable heterogeneity exists among studies. Specifically, there were variations in key treatment parameters across the included trials, such as the dosing (e.g., induction and maintenance doses of anti-CD38 antibodies), treatment duration, and administration schedules of the combination regimens. This clinical heterogeneity, often insufficiently reported, may introduce unmeasured confounding into the network meta-analysis, potentially affecting the precision of the estimated comparative effects and ranking probabilities. Although subgroup analyses were performed, residual confounding factors may persist. Nevertheless, these limitations do not undermine the reliability of the study’s primary conclusions. (3) As a spontaneous reporting system, FAERS is subject to reporting bias, underreporting, and incomplete information. (4) It does not allow for direct calculation of incidence rates or establishment of definitive causal relationships. (5) The lack of detailed clinicopathological information precluded subgroup analyses based on patient baseline characteristics.

Despite these limitations, our study possesses notable strengths. To our knowledge, this is the first integrated analysis that simultaneously employs a Bayesian network meta-analysis of RCTs and a large-scale real-world pharmacovigilance study to evaluate frontline NDMM regimens. This dual approach provides not only a rigorous hierarchy of comparative efficacy but also uncovers clinically significant and previously underappreciated safety signals (e.g., specific opportunistic infections and cardiac events) that might be overlooked in controlled trials. Thus, our findings offer a uniquely comprehensive perspective for balancing efficacy and safety in clinical decision-making.

To address these limitations and translate our findings into future research and practice, we propose the following directions: Firstly, future randomized trials should aim for standardized reporting of dosing and treatment schedules to facilitate more precise meta-analyses. Head-to-head comparisons among the most promising regimens (e.g., D_VRd vs. Isa_VRd) are ultimately needed. Secondly, the potential safety signals identified, particularly severe infections, require prospective validation in studies with detailed clinical data. Finally, clinicians should consider implementing vigilant monitoring strategies for patients on high-efficacy regimens like D_VRd, paying particular attention to the risk of late-onset toxicities suggested by our pharmacovigilance data.

## 4. Materials and Methods

### 4.1. Network Meta-Analysis

The systematic review was reported in accordance with the Preferred Reporting Items for Systematic Reviews and Meta-Analyses (PRISMA) 2020 statement. The completed PRISMA checklist is provided as a Supplementary File. The study protocol was registered with The International Prospective Register of Systematic Reviews (PROSPERO-CRD420251114034).

#### 4.1.1. Literature Search Strategy

A systematic literature search was conducted across four major databases: PubMed, Embase, Web of Science, and the Cochrane Library. The search encompassed records from the inception of each database up to April 2025. The search strategy combined Medical Subject Headings (MeSH)/Emtree terms with free-text keywords to ensure comprehensive coverage. Using PubMed as an example, the detailed search strategy is provided in Supplementary Table S4.

#### 4.1.2. Inclusion and Exclusion Criteria

Studies were included if they met all of the following criteria: adult patients (≥18 years) with NDMM; evaluated doublet, triplet, or quadruplet induction regimens comprising at least one agent from the following classes—PIs, IMiDs, or anti-CD38 monoclonal antibodies—in combination with dexamethasone; reported at least one of the following clinical outcomes: ORR, CR, PFS, OS, MRD negativity rate, grade ≥ 3 AEs, hematologic toxicity, neurotoxicity, infection risk, or discontinuation due to AEs; and were published RCT. Studies were excluded if they focused on relapsed/refractory or smoldering myeloma, utilized non-randomized designs, evaluated monotherapy, employed core regimens beyond the specified drug classes, or lacked essential data.

#### 4.1.3. Data Extraction and Quality Assessment

Data were systematically extracted using a pre-designed form, which primarily collected information on study characteristics, patient demographics, and clinical outcomes. The extraction process was conducted independently by two investigators. Any discrepancies were resolved through discussion or, if necessary, by arbitration from a third investigator. The quality and risk of bias of the included studies were assessed using version 2 of the Cochrane Risk of Bias tool (Cochrane Collaboration, London, UK) for randomized trials.

#### 4.1.4. Statistical Analysis

All statistical analyses were performed using R software (version 4.3.3; R Foundation for Statistical Computing, Vienna, Austria) with relevant packages. To ensure model convergence and stability, Markov chains were configured with four chains, each iterated 50,000 times, with the initial 20,000 iterations designated as the burn-in period. For efficacy and safety outcomes, ORs or HRs with their 95% confidence intervals (CIs) were calculated. The SUCRA values were also estimated. A network evidence graph was generated using STATA (version 16.0; Stata Corp LLC, College Station, TX, USA). Inconsistency tests were performed for outcomes forming closed loops; if the *p*-value exceeded 0.05, a consistency model was applied; otherwise, an inconsistency model was used. Publication bias was assessed via funnel plots and Egger’s test.

### 4.2. Signal Generation

#### 4.2.1. Data Source

The study utilized quarterly updated data from the FAERS database, encompassing all records from the first quarter of 2015 through the second quarter of 2025 (42 quarters total). The analysis encompassed core data tables, including demographic information (DEMO), adverse events (REAC), drug/biologic information (DRUG), therapy administration dates (THER), indications for use (INDI), patient outcomes (OUTC), and reporter sources (RPSR). All tables were integrated and linked through the unique identifier field PRIMARYID.

#### 4.2.2. Data Acquisition and Preprocessing

Due to issues such as duplicate reports, diverse data sources, and partial data gaps within the FAERS database, we conducted systematic data cleaning in accordance with the recommendations of the U.S. FDA [61]. We sorted the CASE_ID, FDA_DT, and PRIMARYID fields in the DEMO table. For reports with identical CASE_IDs, we retained the record with the largest FDA_DT value; When both CASE_ID and FDA_DT were identical, the record with the largest PRIMARYID was retained. This process established a unique report set for analysis [62]. All adverse events were described and classified using the Preferred Terms (PT) from the 28.1 version of the Medical Dictionary for Regulatory Activities (MedDRA). Reports were included if daratumumab, bortezomib, lenalidomide, or dexamethasone was designated as the “Primary Suspect (PS)” drug, and the remaining three drugs were concurrently documented.

#### 4.2.3. Disproportionality Analysis

Signal detection was performed using the ROR and the BCPNN algorithms. For the ROR method, a potential signal was generated if the number of reports for a specific drug-event combination met the threshold of a ≥ 3 and the lower limit of the 95% CI for the ROR exceeded 1. A higher value for this lower limit indicates a stronger association strength between the drug and the event [63]. For the BCPNN method, a potential signal was suggested when the lower limit of the 95% CI for the Information Component (IC) was greater than 0 [64]. A valid adverse event signal is identified when both algorithms yield positive results.

## 5. Conclusions

Quadruplet regimens containing anti-CD38 monoclonal antibodies demonstrate significant advantages in PFS and deep molecular responses. However, notable differences in efficacy and safety profiles exist among the various combination therapies. Real-world data mining has, for the first time, systematically identified potential severe safety signals associated with the D_VRd regimen that are not documented in the prescribing information. These findings underscore the necessity of prioritizing individualized risk assessment and long-term safety management alongside the pursuit of deep therapeutic responses.

## Figures and Tables

**Table 1:** Basic Characteristics of Included Literature.

Study	Year	Intervention	Control	TE/TIE	N	Age	%F	Outcomes
GMMG-HD7	2024	Isa_VRd	VRd	TE	660	60	37.5	①②③④⑥⑦⑧⑨⑩
CASSIOPEIA	2024	D_VTd	VTd	TE	1085	58	41.5	①②③④⑤⑥⑦⑧⑨⑩
PERSEUS	2024	D_VRd	VRd	TE	709	60	41.3	①②③④⑥⑦⑧⑨⑩
GRIFFIN	2023	D_VRd	VRd	TE	207	60	43	①②③④⑤⑥⑦⑧⑨⑩
IsKia	2023	Isa_KRd	KRd	TE	302	61	NA	①②③⑥⑦⑧⑨⑩
Lalit	2019	VRd	VCd	TE	125	58	41.6	①②③④⑤⑥⑦⑧⑨⑩
IFM2013-04	2016	VTd	VCd	TE	340	59	38	②③④⑤⑥⑦⑧⑨⑩
Heinz	2015	VTdC	VTd	TE	98	57	48	①②④⑤⑥⑦⑧⑨⑩
GIMEMA	2020	VTd	Td	TE	480	58	42.5	①②③④⑤⑥⑦⑧⑨⑩
IFM2007-02	2011	Vd	VTd	TE	199	57	42.2	①②③④⑤⑥⑦⑧⑨⑩
ENDURANCE	2020	KRd	VRd	TE	1087	65	41	①②③④⑤⑦⑧⑨⑩
SWOG S0777	2017	VRd	Rd	TIE	525	63	46	①②④⑤⑥⑦⑧⑨⑩
SWOG-1211	2021	Elo_VRd	VRd	TIE	100	66	40	①②④⑤⑥⑦⑧⑨⑩
EMN20	2023	Rd	KRd	TIE	82	73	NA	①②③⑤⑦⑧⑩
Saad	2025	D_VRd	VRd	TIE	395	70	49.8	①②③④⑤⑥⑦⑧⑨⑩
ALCYONE	2025	D_VMP	VMP	TIE	706	71	54	①②③④⑤⑥⑦⑧⑨⑩
OCTANS	2025	D_VMP	VMP	TIE	220	70	40	①②③④⑤⑥⑦⑧⑨⑩
MAIA	2025	D_Rd	Rd	TIE	737	73	32	①②③④⑤⑥⑦⑧⑨⑩
AMaRC 03-16	2024	D_VCd	VCd	TIE	121	75	31	①②③④⑤⑥⑦⑧⑨⑩
AGMT-MM02	2024	KTd	KRd	TIE	122	75	NA	①②③④⑤⑦⑧⑨⑩
BENEFIT/IFM	2024	Isa_VRd	Isa_Rd	TIE	270	70	46	①②③④⑤⑥⑦⑧⑩
REAL	2023	Rd	VMP	TIE	231	77	NA	①②④⑤⑥⑦⑧⑨⑩
IFM2017-03	2022	D_Rd	Rd	TIE	295	68	NA	①②③④⑤⑥⑦⑧⑨⑩
TOURMALINE	2020	IRd	Rd	TIE	705	73	NA	①②③④⑤⑥⑦⑧⑨⑩
GMMG-HD6	2021	Elo _VRd	VRd	TE	559	59	NA	①②③④⑤⑥⑦⑧⑨⑩
GEM2005	2014	VTP	VMP	TIE	260	73	50	①②④⑤⑥⑦⑧⑨⑩
UPFRONT	2013	VTd\VMP	Vd	TIE	502	73	39	①②④⑥⑦⑧⑨⑩
GEM2017FIT	2023	KRd\D_KRd	VMP	TIE	462	72	39	①②③④⑤⑥⑦⑧⑨⑩
EVOLUTION	2012	VRdC VRd	VCd	TIE	140	61	43.6	①②③⑥⑦⑧⑨⑩
PETHEMA/GEM	2012	VTd	Td\VMP	TE	386	57	39	①②③⑥⑦⑧⑨⑩
Mookerjee	2017	VRd	Rd	TIE	144	56	32.5	①②③④⑤⑥⑦⑧⑨⑩
IMROZ	2024	Isa_VRd	VRd	TIE	446	NA	NA	①②③④⑤⑥⑦⑧⑨⑩
Kohmei	2020	Elo_Rd	Rd	TIE	82	73	34.1	②④⑤⑥⑦⑧⑩

Abbreviations: NA, not available; D, daratumumab; Isa, isatuximab; Elo, elotuzumab; V, bortezomib; M, melphalan; P, prednisone; K, carfilzomib; R, lenalidomide; d, dexamethasone; T, thalidomide; C, cyclophosphamide; I, ixazomib; F, Female patients; TE, transplant-eligible; TIE, transplant-ineligible. Outcome measures: ①, overall response rate (ORR); ②, complete response rate (CR); ③, minimal residual disease (MRD) negativity rate; ④, PFS; ⑤, overall survival (OS); ⑥, grade ≥ 3 adverse events (AEs); ⑦, discontinuation rate due to adverse events; ⑧, hematological toxicity; ⑨, neurotoxicity; ⑩, infection risk. Note: Values presented as reported in the original publications. The symbols in the “Outcomes” column indicate which efficacy and safety endpoints were reported in each respective study.

**Table 2:** Top 20 Most Frequently Reported Adverse Events for the D_VRd Regimen.

Adverse Event (PT)	Case Count	ROR (95%Cl)	IC (IC025)
Peripheral neuropathy	236	1.43 (1.26–1.63)	0.5 (0.31)
Neutropenia	220	1.95 (1.71–2.24)	0.94 (0.74)
Infectious pneumonia	217	0.83 (0.72–0.95)	−0.27 (−0.47)
Fatigue	208	0.6 (0.53–0.69)	−0.7 (−0.91)
Diarrhea	198	0.56 (0.48–0.64)	−0.82 (−1.03)
Anemia	178	1.97 (1.7–2.29)	0.95 (0.73)
Thrombocytopenia	169	2.03 (1.74–2.37)	1 (0.77)
Rash	161	0.84 (0.72–0.98)	−0.25 (−0.48)
Infusion-related reaction	142	6.26 (5.28–7.43)	2.55 (2.3)
Sepsis	103	1.94 (1.6–2.37)	0.94 (0.65)
Polyneuropathy	91	9.35 (7.53–11.62)	3.08 (2.77)
Febrile neutropenia	87	3.03 (2.45–3.76)	1.56 (1.25)
Acute kidney injury	81	2.03 (1.63–2.54)	1 (0.68)
White blood cell count decreased	75	0.53 (0.42–0.66)	−0.9 (−1.24)
Pancytopenia	67	1.72 (1.35–2.2)	0.77 (0.41)
Infection	67	1.05 (0.82–1.34)	0.07 (−0.28)
Herpes zoster	61	2.37 (1.83–3.05)	1.22 (0.84)
Urinary tract infection	55	0.87 (0.67–1.14)	−0.19 (−0.58)
Atrial fibrillation	53	1.2 (0.91–1.57)	0.26 (−0.14)
Neutrophil count decreased	53	1.32 (1.01–1.73)	0.39 (0)

Note: D_VRd: daratumumab, bortezomib, lenalidomide, and dexamethasone. PT, Preferred Term; ROR, Reporting Odds Ratio; IC, information component; IC025, lower bound of the 95% credibility interval for the IC. Data source: FDA Adverse Event Reporting System (FAERS), Q1 2015–Q2 2025.

**Table 3:** Distribution of Unlabeled Safety Signals for the D_VRd Regimen.

System Organ Class (SOC)	Signal Count	Proportion (%)	Unlabeled Signals Count
Blood and lymphatic system disorders	721	28.57	0
Nervous system disorders	408	16.16	5
Infections and infestations	277	10.97	3
General disorders and administration site conditions	174	6.89	4
Injury, poisoning, and procedural complications	142	5.63	0
Renal and urinary disorders	118	4.68	4
Metabolism and nutrition disorders	105	4.16	3
Skin and subcutaneous tissue disorders	94	3.72	5
Respiratory, thoracic, and mediastinal disorders	88	3.49	4
Gastrointestinal disorders	59	2.34	4
Investigations	59	2.34	5
Hepatobiliary disorders	57	2.26	5
Neoplasms benign, malignant, and unspecified (incl cysts and polyps)	56	2.22	4
Musculoskeletal and connective tissue disorders	52	2.06	3
Immune system disorders	51	2.02	4
Cardiac disorders	27	1.07	5
Psychiatric disorders	20	0.79	3
Vascular disorders	16	0.63	3
Total	2524	100.00	64

## Data Availability

The data supporting the findings of this network meta-analysis were deposited in the Figshare repository under the accession DOI [10.6084/m9.figshare.30010282].

## Supplementary Materials

The following supporting information can be downloaded at: https://www.mdpi.com/article/10.3390/ph19010018/s1. Table S1: Model Fit and Heterogeneity Statistics for Different Outcomes. Table S2: Baseline Characteristics of Reported Adverse Events. Table S3: Top 20 Adverse Event Signals Ranked by Signal Strength for the Daratumumab Group. Table S4: PubMed Database Search Strategy. Figure S1: Risk of bias assessment summary for the included randomized controlled trials. Figure S2: League Plot for the NMA of MRD Negativity Rate. Figure S3: League Plot for the NMA of PFS. Figure S4: NMA of ORR. Figure S5: League Plot for the NMA of ORR. Figure S6: NMA of CR. Figure S7: League Plot for the NMA of CR. Figure S8: NMA of OS. Figure S9: League Plot for the NMA of OS. Figure S10: Subgroup Analysis of ORR in TE and TIE Populations. Figure S11: Subgroup Analysis of CR in TE and TIE Populations. Figure S12: Subgroup Analysis of PFS in TE and TIE Populations. Figure S13: Subgroup Analysis of OS in TE and TIE Populations. Figure S14: NMA of Grade ≥ 3AEs. Figure S15: League Plot for the NMA of Grade ≥ 3AEs. Figure S16. NMA of Treatment Discontinuation Due to Adverse Events. Figure S17: League Plot for the NMA of Treatment Discontinuation Due to Adverse Events. Figure S18: NMA of Hematologic Toxicity. Figure S19: League Plot for the NMA of Hematologic Toxicity. Figure S20: NMA of Infection Risk. Figure S21: League Plot for the NMA of Infection Risk. Figure S22: NMA of Neurotoxicity. Figure S23: League Plot for the NMA of Neurotoxicity. Figure S24: FAERS Database Screening Flowchart. Figure S25: Distribution of Adverse Events by SOC.

## Abbreviations

The following abbreviations are used in this manuscript:

AE	Adverse Event
CR	Complete Response
FAERS	FDA Adverse Event Reporting System
NMA	Network Meta-Analysis
ORR	Objective Response Rate
OS	Overall Survival
PFS	Progression-Free Survival
SOC	System Organ Class
SUCRA	Surface Under the Cumulative Ranking Curve
