# Supplementary material for: Efficacy and Safety of Newly Diagnosed Multiple Myeloma Combination Therapies: A Systematic Review Integrating Network Meta-Analysis and Real-World Vigilance Study
Source: Pharmaceuticals (Basel). 2025 Dec 21;19(1):18. doi: 10.3390/ph19010018 (PMC12845409; doi:10.3390/ph19010018)
Supplement: Supplementary file 1 [file pharmaceuticals-19-00018-s001.zip › pharmaceuticals-4053197-supplementary.pdf]

Supplementary Materials

List of Abbreviations:

1. Treatment Regimens & Components:

   D, daratumumab

   Isa, isatuximab

   Elo, elotuzumab

   V, bortezomib

   M, melphalan

   P, prednisone

   K, carfilzomib

   R, lenalidomide

   d, dexamethasone

   T, thalidomide

   C, cyclophosphamide

   I, ixazomib

2.Methodological Terms:

   NMA, network meta-analysis

   SUCRA, surface under the cumulative ranking curve

   ROR, reporting odds ratio

   HR, hazard ratio

   OR, odds ratio

   CI, confidence interval

3.Outcome Measures:

   ORR, overall response rate

   CR, complete response

   PFS, progression-free survival

   OS, overall survival

   MRD, minimal residual disease

   AE, adverse event

4.Other Key Terms:

   FAERS, FDA Adverse Event Reporting System

   SOC, system organ class

   PT, preferred term (MedDRA terminology)

   TE, transplant-eligible

   TIE, transplant-ineligible

Table S1. Model Fit and Heterogeneity for Different Outcomes.

Outcome	Mode	DIC	I^2
ORR	Consistency	120.45	0%
	Inconsistency	121.75	0%
MRD negativity	Consistency	95.81	0%
	Inconsistency	98.98	0%
PFS	Consistency	48.34	0%
	Inconsistency	52.27	0%
OS	Consistency	47.95	0%
	Inconsistency	48.58	0%
CR	Consistency	120.87	0%
	Inconsistency	124.97	0%
Grade ≥3 AEs	Consistency	111.63	0%
	Inconsistency	115.64	0%
Hematologic toxicity	Consistency	121.49	0%
	Inconsistency	122.19	0%
Infection risk	Consistency	121.08	0%
	Inconsistency	121.36	0%
Neurotoxicity	Consistency	107.97	0%
	Inconsistency	108.18	0%
Treatment discontinuation due to AEs	Consistency	122.23	0%
	Inconsistency	126.05	0%

Note: DIC, deviance information criterion; I^2, I-squared statistic (measure of heterogeneity); "Consistency" and "Inconsistency" refer to the corresponding models in network meta-analysis.

Table S2. Baseline Characteristics of Reported Adverse Events.

Characteristics	D_VRd regimen (N=3,861) n (%)
**Gender**	
Male	1,755 (45.5)
Female	1,470 (38.1)
Unknown	636 (16.5)
**Age Group (Years)**	
< 18	78 (2.0)
18 - 65	1,281 (33.2)
65 - 85	1,497 (38.8)
> 85	33 (0.9)
Unknown	972 (25.2)
**Reporter**	
Physician	1,989 (51.5)
Pharmacist	365 (9.5)
Other Health Professional	1,153 (29.9)
Consumer	305 (7.9)
Unknown	49 (1.2)
**Outcomes**	
Death	494 (12.8)
Life-Threatening	186 (4.8)
Hospitalization-Prolonged	1,086 (28.1)
Disability	13 (0.4)
Other	2,082 (53.9)

Note: N, total number of cases; n, number of cases within a category. Data are presented as n (%). This analysis specifically reports the baseline characteristics of patients with adverse events associated with the D_VRd regimen.

Table S3. Top 20 Adverse Event Signals by Strength for the D_VRd Regimen.

**Adverse Event (PT)**	**Case Count**	**ROR (95%Cl)**	**IC(IC025)**
Pneumomediastinum	14	109.09 (49.51-240.34)	4.66
Pulmonary mucormycosis	8	171.34 (51.59-569.08)	4.6
BK virus infection	28	54.61 (33.99-87.74)	4.44
HCoV-OC43 infection	6	171.31 (42.84-685.06)	4.43
Bacterial laryngitis	4	342.56 (38.28-3065.17)	4.37
Rheumatic disease	7	85.66 (30.04-244.26)	4.17
Cytomegalovirus enteritis	4	171.28 (31.37-935.23)	4.16
Acinetobacter bacteraemia	3	256.89 (26.72-2469.95)	4.09
Hyperlipasaemia	3	256.89 (26.72-2469.95)	4.09
Viral haemorrhagic cystitis	6	85.65 (27.62-265.62)	4.08
Acinetobacter infection	9	55.08 (23.83-127.27)	4
Haemorrhagic cystitis	20	29.07 (17.5-48.28)	3.75
Enterovirus infection	7	46.12 (18.4-115.63)	3.72
Otitis externa	7	42.83 (17.28-106.14)	3.66
Respiratory virus test positive	4	57.09 (16.11-202.35)	3.56
Cytomegalovirus gastroenteritis	4	57.09 (16.11-202.35)	3.56
Bronchopulmonary aspergillosis	35	19.02 (13.18-27.44)	3.45
Toxic shock syndrome	3	64.22 (14.37-286.99)	3.45
Clostridial sepsis	5	38.93 (13.52-112.06)	3.39
Beta-haemolytic streptococcal infection	5	35.69 (12.57-101.31)	3.31

Note: IC, information component; IC025, lower bound of the 95% credibility interval for the IC. Signals are ranked by the strength of association (IC value). This table presents signals specifically associated with the D_VRd regimen.

Table S4. PubMed Database Search Strategy.

Number	Search Query
#1	"Multiple Myeloma"[MeSH Terms] OR "multiple myelomas"[Title/Abstract] OR (("Multiple Myeloma"[MeSH Terms] OR ("Multiple"[All Fields] AND "Myeloma"[All Fields]) OR "Multiple Myeloma"[All Fields] OR "Myeloma"[All Fields] OR "Myelomas"[All Fields] OR "myeloma s"[All Fields]) AND "Multiple"[Title/Abstract]) OR "myeloma plasma cell"[Title/Abstract] OR "myeloma plasma cell"[Title/Abstract] OR "myelomas plasma cell"[Title/Abstract] OR "plasma cell myeloma"[Title/Abstract] OR "plasma cell myelomas"[Title/Abstract] OR "Myeloma-Multiple"[Title/Abstract] OR "Myeloma-Multiple"[Title/Abstract] OR "Myeloma-Multiple"[Title/Abstract] OR "plasma cell myeloma"[Title/Abstract] OR (("cells"[MeSH Terms] OR "cells"[All Fields] OR "Cell"[All Fields]) AND "myeloma plasma"[Title/Abstract]) OR (("cells"[MeSH Terms] OR "cells"[All Fields] OR "Cell"[All Fields]) AND "myelomas plasma"[Title/Abstract]) OR "myelomas plasma cell"[Title/Abstract] OR "plasma cell myelomas"[Title/Abstract] OR "kahler disease"[Title/Abstract] OR (("Disease"[MeSH Terms] OR "Disease"[All Fields] OR "diseases"[All Fields] OR "disease s"[All Fields] OR "diseased"[All Fields]) AND "Kahler"[Title/Abstract]) OR "Myelomatosis"[Title/Abstract] OR "Myelomatoses"[Title/Abstract]
#2	"Proteasome Inhibitors"[MeSH Terms] OR "inhibitors proteasome"[Title/Abstract] OR ((("proteasomal"[All Fields] OR "proteasomally"[All Fields] OR "proteasome endopeptidase complex"[Supplementary Concept] OR "proteasome endopeptidase complex"[All Fields] OR "Proteasome"[All Fields] OR "proteasome endopeptidase complex"[MeSH Terms] OR ("Proteasome"[All Fields] AND "endopeptidase"[All Fields] AND "Complex"[All Fields]) OR "proteasomes"[All Fields] OR "proteasome s"[All Fields] OR "proteasomic"[All Fields]) AND ("endopeptidases"[Supplementary Concept] OR "endopeptidases"[All Fields] OR "endopeptidase"[All Fields] OR "endopeptidases"[MeSH Terms])) AND "complex inhibitors"[Title/Abstract]) OR "proteasome inhibitor"[Title/Abstract] OR "inhibitor proteasome"[Title/Abstract] OR "Bortezomib"[Title/Abstract] OR "Velcade"[Title/Abstract] OR "Carfilzomib"[Title/Abstract] OR "Kyprolis"[Title/Abstract] OR "Ixazomib"[Title/Abstract] OR "Ninlaro"[Title/Abstract] OR "Marizomib"[Title/Abstract] OR "salinosporamide a"[Title/Abstract]

#3	"Immunomodulation"[MeSH Terms] OR "Immunomodulations"[Title/Abstract] OR "immunomodulatory therapy"[Title/Abstract] OR "immunomodulatory therapies"[Title/Abstract] OR "therapies immunomodulatory"[Title/Abstract] OR "therapy immunomodulatory"[Title/Abstract] OR "Thalidomide"[Title/Abstract] OR "Sedoval"[Title/Abstract] OR "Thalomid"[Title/Abstract] OR "Lenalidomide"[Title/Abstract] OR "Revlimid"[Title/Abstract] OR "Revimid"[Title/Abstract] OR "Pomalidomide"[Title/Abstract] OR "actimid"[Title/Abstract] OR "Pomalyst"[Title/Abstract] OR "Imnovid"[Title/Abstract]
#4	"antibodies, monoclonal"[MeSH Terms] OR "monoclonal antibody"[Title/Abstract] OR "antibody monoclonal"[Title/Abstract] OR "monoclonal antibodies"[Title/Abstract] OR "Daratumumab"[Title/Abstract] OR "Darzalex"[Title/Abstract] OR "Isatuximab"[Title/Abstract] OR "Sarclisa"[Title/Abstract] OR "isatuximab-irfc"[Title/Abstract] OR "Elotuzumab"[Title/Abstract] OR "Empliciti"[Title/Abstract]
#5	"Randomized Controlled Trials as Topic"[MeSH Terms] OR "randomized controlled trial"[Publication Type]
#6	（（#2 OR #3 OR #4）AND #1）AND #5

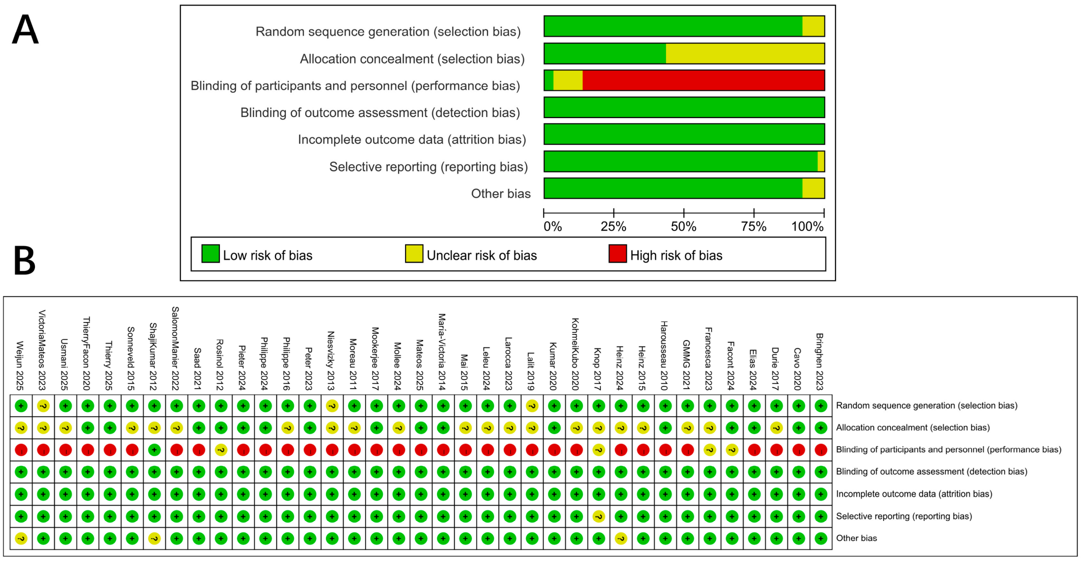

Figure S1. Risk of bias assessment summary for the included randomized controlled trials.
(A) Risk of bias graph presenting the proportion of studies with each judgment across all domains. (B) Risk of bias table showing the detailed judgment for each domain in each study.

D_KRd																				
**3.46 (1.38, 9.15)**	D_Rd																			
3.14 (0.95, 10.49)	0.9 (0.24, 3.37)	D_VCd																		
1.41 (0.69, 2.86)	0.41 (0.15, 1.12)	0.45 (0.14, 1.51)	D_VMP																	
1.51 (0.78, 2.9)	**0.44 (0.19, 0.98)**	0.47 (0.16, 1.54)	1.07 (0.49, 2.34)	D_VRd																
**3 (1.35, 6.53)**	0.86 (0.3, 2.4)	0.94 (0.31, 2.93)	2.12 (0.97, 4.6)	1.98 (0.89, 4.3)	D_VTd															
**3.63 (1.71, 7.68)**	1.04 (0.43, 2.52)	1.15 (0.36, 3.89)	**2.57 (1.1, 6.04)**	**2.41 (1.42, 4.09)**	1.21 (0.52, 2.88)	Elo_VRd														
**7.33 (2.71, 20.27)**	**2.11 (1.16, 3.86)**	2.32 (0.59, 9.18)	**5.21 (1.79, 15)**	**4.88 (2.05, 11.75)**	2.45 (0.82, 7.3)	2.03 (0.79, 5.22)	IRd													
1.82 (0.82, 3.97)	0.52 (0.19, 1.39)	0.57 (0.16, 2.04)	1.29 (0.52, 3.15)	1.21 (0.58, 2.48)	0.61 (0.24, 1.54)	0.5 (0.22, 1.11)	**0.25 (0.09, 0.69)**	Isa_KRd												
**9.4 (3.95, 22.64)**	**2.69 (1, 7.26)**	2.98 (0.85, 10.78)	**6.69 (2.56, 17.29)**	**6.22 (3.12, 12.54)**	**3.15 (1.2, 8.36)**	**2.59 (1.19, 5.63)**	1.27 (0.45, 3.66)	**5.17 (2.05, 13.15)**	Isa_Rd											
**2.83 (1.45, 5.67)**	0.82 (0.35, 1.87)	0.9 (0.29, 2.87)	2.02 (0.92, 4.5)	**1.89 (1.26, 2.85)**	0.95 (0.43, 2.13)	0.78 (0.45, 1.35)	**0.39 (0.16, 0.95)**	1.56 (0.76, 3.28)	**0.3 (0.17, 0.53)**	Isa_VRd										
**4.25 (2.52, 7.13)**	1.23 (0.54, 2.67)	1.34 (0.44, 4.15)	**3.01 (1.54, 5.94)**	**2.82 (1.81, 4.33)**	1.41 (0.69, 2.95)	1.17 (0.67, 2.04)	0.58 (0.24, 1.36)	**2.33 (1.31, 4.21)**	**0.45 (0.22, 0.92)**	1.5 (0.95, 2.33)	KRd									
**6.64 (2.4, 19.13)**	1.91 (0.58, 6.4)	2.12 (0.5, 8.74)	**4.7 (1.55, 14.91)**	**4.38 (1.67, 12.09)**	2.22 (0.73, 7.06)	1.81 (0.65, 5.41)	0.9 (0.26, 3.17)	**3.64 (1.29, 10.91)**	0.71 (0.23, 2.25)	2.33 (0.87, 6.49)	1.56 (0.66, 3.92)	KTd								
**10.11 (4.29, 24.57)**	**2.91 (2.03, 4.18)**	3.21 (0.91, 11.64)	**7.16 (2.77, 18.89)**	**6.71 (3.27, 14.14)**	**3.39 (1.29, 9.04)**	**2.8 (1.25, 6.35)**	1.38 (0.86, 2.24)	**5.57 (2.26, 14.16)**	1.08 (0.43, 2.75)	**3.56 (1.7, 7.55)**	**2.38 (1.2, 4.93)**	1.53 (0.48, 4.77)	Rd							
**12.29 (5.63, 26.53)**	**3.53 (1.25, 9.75)**	**3.87 (1.29, 12.48)**	**8.73 (4.18, 18.31)**	**8.15 (3.76, 17.53)**	**4.11 (2.37, 7.14)**	**3.39 (1.45, 7.88)**	1.67 (0.57, 4.96)	**6.78 (2.73, 16.92)**	1.31 (0.5, 3.4)	**4.32 (1.98, 9.39)**	**2.9 (1.44, 5.83)**	1.86 (0.59, 5.54)	1.21 (0.46, 3.16)	Td						
**9.15 (4.35, 19.66)**	**2.65 (1.01, 6.81)**	**2.9 (1.19, 7.4)**	**6.49 (2.98, 14.19)**	**6.12 (3.1, 11.77)**	**3.08 (1.62, 5.85)**	**2.53 (1.2, 5.36)**	1.25 (0.46, 3.43)	**5.07 (2.16, 12.09)**	0.98 (0.41, 2.33)	**3.23 (1.64, 6.35)**	**2.16 (1.15, 4.06)**	1.38 (0.47, 4.02)	0.91 (0.37, 2.19)	0.75 (0.39, 1.45)	VCd					
**18.11 (5.16, 67.69)**	**5.16 (1.28, 22.82)**	**5.75 (1.26, 28.11)**	**12.79 (3.69, 47.07)**	**12.04 (3.42, 44.76)**	**6.06 (2.06, 19.67)**	**4.97 (1.36, 19.35)**	2.44 (0.58, 11.09)	**10.05 (2.56, 40.44)**	1.94 (0.5, 7.93)	**6.41 (1.78, 23.78)**	**4.27 (1.28, 15.26)**	2.73 (0.6, 12.64)	1.77 (0.46, 7.53)	1.47 (0.48, 4.89)	1.96 (0.61, 7.02)	Vd				
2.15 (0.8, 5.91)	0.62 (0.2, 1.91)	0.69 (0.19, 2.44)	1.53 (0.53, 4.43)	1.44 (0.59, 3.46)	0.73 (0.26, 2.03)	0.6 (0.23, 1.54)	**0.3 (0.09, 0.94)**	1.2 (0.42, 3.39)	**0.23 (0.08, 0.67)**	0.76 (0.31, 1.85)	0.51 (0.21, 1.23)	0.33 (0.09, 1.12)	**0.21 (0.07, 0.62)**	**0.18 (0.06, 0.49)**	**0.24 (0.1, 0.56)**	**0.12 (0.03, 0.5)**	VRdC			
**7.66 (4.5, 13.36)**	2.23 (0.87, 5.54)	2.42 (0.81, 7.63)	**5.43 (3.51, 8.58)**	**5.1 (2.71, 9.57)**	**2.57 (1.38, 4.89)**	**2.12 (1.04, 4.35)**	1.05 (0.4, 2.78)	**4.24 (1.95, 9.28)**	0.82 (0.35, 1.91)	**2.71 (1.42, 5.12)**	**1.81 (1.09, 2.98)**	1.16 (0.41, 3.19)	0.76 (0.32, 1.75)	0.63 (0.35, 1.14)	0.84 (0.44, 1.59)	0.43 (0.12, 1.35)	**3.57 (1.35, 9.27)**	VMP		
**4.83 (2.67, 8.88)**	1.4 (0.64, 3)	1.53 (0.52, 4.74)	**3.44 (1.68, 7.21)**	**3.21 (2.46, 4.26)**	1.62 (0.79, 3.41)	1.33 (0.85, 2.11)	0.66 (0.29, 1.52)	**2.67 (1.38, 5.27)**	**0.52 (0.27, 0.98)**	**1.71 (1.25, 2.32)**	1.14 (0.83, 1.61)	0.73 (0.28, 1.85)	**0.48 (0.24, 0.93)**	**0.39 (0.19, 0.81)**	**0.53 (0.29, 0.97)**	**0.27 (0.07, 0.91)**	2.23 (0.97, 5.2)	0.63 (0.36, 1.11)	VRd	
**5.45 (2.68, 11.06)**	1.57 (0.58, 4.11)	1.72 (0.61, 5.03)	**3.86 (1.92, 7.76)**	**3.63 (1.77, 7.22)**	**1.83 (1.28, 2.6)**	1.51 (0.68, 3.28)	0.74 (0.27, 2.08)	**3 (1.27, 7.2)**	0.58 (0.23, 1.42)	1.92 (0.94, 3.88)	1.29 (0.68, 2.42)	0.82 (0.27, 2.4)	0.53 (0.22, 1.32)	0.44 (0.29, 0.67)	0.59 (0.35, 1.01)	0.3 (0.1, 0.83)	2.51 (0.96, 6.55)	0.71 (0.41, 1.19)	1.13 (0.58, 2.12)	VTd

Figure S2. League Plot for the NMA of MRD Negativity Rate.

League table presenting ORs with 95% CIs for all pairwise comparisons. Each cell compares the column-defining intervention against the row-defining intervention. An OR with a 95% CI not including 1 indicates a statistically significant difference (P < 0.05). An OR > 1 favors the column-defining intervention. Treatment regimen abbreviations are defined in the List of Abbreviations.

D_KRd																					
0.67 (0.38, 1.2)	D_Rd																				
0.59 (0.29, 1.19)	0.87 (0.49, 1.56)	D_VCd																			
0.81 (0.49, 1.37)	1.2 (0.77, 1.93)	1.38 (0.75, 2.53)	D_VMP																		
1.13 (0.64, 2.03)	**1.68 (1.12, 2.54)**	**1.93 (1.1, 3.39)**	1.4 (0.87, 2.21)	D_VRd																	
0.87 (0.48, 1.63)	1.3 (0.79, 2.15)	1.49 (0.86, 2.58)	1.08 (0.67, 1.73)	0.77 (0.48, 1.25)	D_VTd																
0.52 (0.22, 1.23)	0.78 (0.39, 1.57)	0.89 (0.38, 2.1)	0.65 (0.3, 1.39)	**0.46 (0.22, 0.97)**	0.6 (0.27, 1.33)	Elo_Rd															
**0.55 (0.32, 0.96)**	0.81 (0.56, 1.2)	0.93 (0.54, 1.61)	0.68 (0.43, 1.05)	**0.48 (0.35, 0.68)**	**0.63 (0.39, 0.99)**	1.05 (0.5, 2.18)	Elo_VRd														
**0.45 (0.25, 0.82)**	**0.67 (0.48, 0.95)**	0.77 (0.42, 1.4)	**0.56 (0.34, 0.9)**	**0.4 (0.26, 0.61)**	**0.52 (0.31, 0.86)**	0.87 (0.42, 1.77)	0.83 (0.55, 1.23)	IRd													
0.52 (0.23, 1.14)	0.76 (0.39, 1.54)	0.88 (0.4, 1.92)	0.63 (0.31, 1.32)	**0.45 (0.24, 0.88)**	0.59 (0.28, 1.23)	0.98 (0.39, 2.49)	0.94 (0.49, 1.8)	1.14 (0.57, 2.35)	Isa_Rd												
0.84 (0.47, 1.49)	1.24 (0.83, 1.89)	1.42 (0.81, 2.5)	1.03 (0.65, 1.65)	0.74 (0.52, 1.06)	0.96 (0.59, 1.56)	1.6 (0.75, 3.41)	1.53 (1.09, 2.14)	**1.85 (1.21, 2.87)**	1.62 (0.92, 2.83)	Isa_VRd											
**0.52 (0.32, 0.86)**	0.78 (0.53, 1.16)	0.89 (0.52, 1.55)	**0.65 (0.43, 0.96)**	**0.46 (0.32, 0.67)**	**0.6 (0.38, 0.94)**	1 (0.48, 2.09)	0.96 (0.68, 1.33)	1.16 (0.76, 1.76)	1.02 (0.52, 1.96)	**0.63 (0.43, 0.9)**	KRd										
**0.25 (0.12, 0.53)**	**0.37 (0.19, 0.74)**	**0.43 (0.2, 0.93)**	**0.31 (0.16, 0.61)**	**0.22 (0.12, 0.43)**	**0.29 (0.14, 0.59)**	0.48 (0.19, 1.21)	**0.46 (0.24, 0.88)**	0.56 (0.28, 1.12)	0.49 (0.21, 1.15)	**0.3 (0.16, 0.58)**	**0.48 (0.28, 0.83)**	KTd									
**0.38 (0.22, 0.64)**	**0.56 (0.45, 0.69)**	0.64 (0.37, 1.09)	**0.46 (0.31, 0.69)**	**0.33 (0.23, 0.47)**	**0.43 (0.27, 0.67)**	0.72 (0.37, 1.39)	**0.68 (0.5, 0.93)**	0.83 (0.64, 1.07)	0.73 (0.37, 1.39)	**0.45 (0.31, 0.63)**	**0.72 (0.51, 0.99)**	1.49 (0.78, 2.83)	Rd								
**0.33 (0.18, 0.63)**	**0.49 (0.29, 0.83)**	**0.56 (0.32, 1)**	**0.41 (0.25, 0.67)**	**0.29 (0.18, 0.48)**	**0.38 (0.27, 0.54)**	0.64 (0.28, 1.42)	**0.6 (0.38, 0.98)**	0.73 (0.43, 1.25)	0.64 (0.3, 1.35)	**0.4 (0.24, 0.66)**	0.63 (0.4, 1.02)	1.31 (0.64, 2.74)	0.88 (0.56, 1.42)	Td							
**0.34 (0.2, 0.6)**	**0.51 (0.34, 0.76)**	**0.58 (0.38, 0.89)**	**0.42 (0.28, 0.64)**	**0.3 (0.21, 0.43)**	**0.39 (0.28, 0.56)**	0.65 (0.31, 1.38)	**0.62 (0.45, 0.88)**	0.76 (0.5, 1.16)	0.67 (0.34, 1.28)	**0.41 (0.28, 0.59)**	**0.65 (0.46, 0.93)**	1.36 (0.71, 2.63)	0.91 (0.65, 1.28)	1.03 (0.71, 1.5)	VCd						
**0.35 (0.2, 0.59)**	**0.51 (0.33, 0.8)**	0.59 (0.34, 1.03)	**0.43 (0.3, 0.6)**	**0.3 (0.2, 0.47)**	**0.4 (0.27, 0.58)**	0.66 (0.31, 1.42)	**0.63 (0.41, 0.97)**	0.76 (0.48, 1.22)	0.67 (0.33, 1.36)	**0.41 (0.26, 0.65)**	**0.66 (0.45, 0.97)**	1.37 (0.7, 2.69)	0.92 (0.63, 1.36)	1.04 (0.69, 1.57)	1.01 (0.71, 1.44)	Vd					
**0.33 (0.21, 0.53)**	**0.48 (0.32, 0.73)**	**0.56 (0.32, 0.97)**	**0.4 (0.32, 0.5)**	**0.29 (0.19, 0.44)**	**0.37 (0.24, 0.57)**	0.62 (0.3, 1.31)	**0.59 (0.4, 0.88)**	0.72 (0.47, 1.1)	0.63 (0.31, 1.26)	**0.39 (0.26, 0.59)**	**0.62 (0.45, 0.87)**	1.29 (0.68, 2.46)	0.87 (0.62, 1.23)	0.98 (0.63, 1.54)	0.95 (0.66, 1.36)	0.94 (0.72, 1.24)	VMP				
**0.55 (0.33, 0.93)**	0.81 (0.59, 1.13)	0.93 (0.57, 1.55)	**0.68 (0.45, 1)**	**0.48 (0.38, 0.63)**	**0.63 (0.42, 0.94)**	1.05 (0.52, 2.13)	1 (0.81, 1.24)	1.21 (0.86, 1.72)	1.07 (0.57, 1.96)	**0.66 (0.51, 0.85)**	1.05 (0.81, 1.36)	**2.18 (1.19, 4)**	**1.46 (1.17, 1.85)**	**1.66 (1.08, 2.54)**	**1.6 (1.23, 2.09)**	**1.59 (1.1, 2.28)**	**1.69 (1.21, 2.33)**	VRd			
**0.53 (0.31, 0.95)**	0.8 (0.51, 1.24)	0.91 (0.56, 1.51)	**0.66 (0.43, 1)**	**0.47 (0.31, 0.72)**	**0.61 (0.49, 0.77)**	1.02 (0.48, 2.19)	0.98 (0.66, 1.46)	1.19 (0.74, 1.88)	1.04 (0.52, 2.07)	**0.64 (0.42, 0.98)**	1.02 (0.69, 1.51)	**2.12 (1.08, 4.19)**	1.43 (0.97, 2.1)	**1.62 (1.23, 2.12)**	**1.57 (1.2, 2.03)**	**1.55 (1.14, 2.11)**	**1.64 (1.14, 2.35)**	0.98 (0.7, 1.36)	VTd		
**0.44 (0.21, 0.96)**	0.66 (0.34, 1.29)	0.76 (0.37, 1.55)	**0.55 (0.28, 1.07)**	**0.39 (0.2, 0.75)**	**0.51 (0.29, 0.89)**	0.85 (0.34, 2.16)	0.81 (0.43, 1.55)	0.98 (0.5, 1.95)	0.86 (0.37, 2.03)	0.53 (0.27, 1.03)	0.84 (0.45, 1.62)	1.76 (0.76, 4.07)	1.18 (0.63, 2.24)	1.33 (0.75, 2.38)	1.29 (0.73, 2.29)	1.28 (0.72, 2.34)	1.36 (0.73, 2.57)	0.81 (0.44, 1.49)	0.83 (0.5, 1.37)	VTdC	
**0.28 (0.17, 0.46)**	**0.41 (0.26, 0.65)**	**0.47 (0.26, 0.84)**	**0.34 (0.25, 0.46)**	**0.24 (0.16, 0.39)**	**0.32 (0.2, 0.49)**	0.53 (0.25, 1.14)	**0.51 (0.33, 0.78)**	**0.61 (0.38, 0.98)**	0.54 (0.26, 1.09)	**0.33 (0.21, 0.52)**	**0.53 (0.36, 0.78)**	1.1 (0.57, 2.15)	0.74 (0.5, 1.09)	0.84 (0.52, 1.33)	0.81 (0.54, 1.2)	0.8 (0.6, 1.07)	0.85 (0.69, 1.05)	**0.5 (0.35, 0.73)**	**0.52 (0.35, 0.76)**	0.63 (0.33, 1.17)	VTP

Figure S3. League Plot for the NMA of PFS.

League table presenting HRs with 95% CIs for all pairwise comparisons. Each cell compares the column-defining intervention against the row-defining intervention. An HR with a 95% CI not including 1 indicates a statistically significant difference (P < 0.05). An HR < 1 favors the column-defining intervention. Treatment regimen abbreviations are defined in the List of Abbreviations.

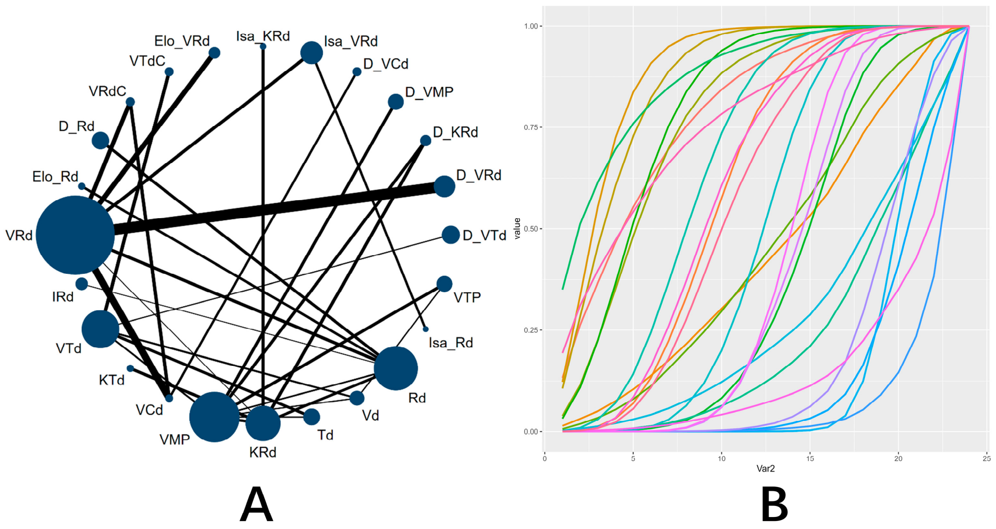

Figure S4. NMA of ORR.

(A) Network plot of treatment comparisons. The size of each node is proportional to the number of participants assigned to that regimen, and the width of each line (edge) is proportional to the number of trials directly comparing the connected regimens. (B) SUCRA plot. A higher SUCRA value (closer to 100%) indicates a greater probability of being the most effective regimen for improving ORR. Treatment regimen abbreviations are defined in the List of Abbreviations.

D_KRd																							
1.66 (0.49, 7.17)	D_Rd																						
2.53 (0.43, 16.95)	1.51 (0.35, 6.36)	D_VCd																					
0.81 (0.24, 3.34)	0.48 (0.2, 1.17)	0.32 (0.07, 1.46)	D_VMP																				
0.85 (0.22, 3.9)	0.51 (0.2, 1.23)	0.34 (0.08, 1.41)	1.06 (0.38, 2.92)	D_VRd																			
1.12 (0.29, 5.29)	0.67 (0.23, 1.92)	0.44 (0.08, 2.31)	1.39 (0.57, 3.35)	1.31 (0.4, 4.38)	D_VTd																		
2.36 (0.49, 12.89)	1.4 (0.45, 4.16)	0.92 (0.16, 5.13)	2.92 (0.8, 10.31)	2.73 (0.77, 9.6)	2.08 (0.52, 8.47)	Elo_Rd																	
1.06 (0.29, 4.83)	0.63 (0.27, 1.49)	0.42 (0.1, 1.74)	1.32 (0.48, 3.5)	1.24 (0.53, 2.97)	0.95 (0.29, 3.06)	0.45 (0.13, 1.63)	Elo_VRd																
2.58 (0.75, 11.18)	1.55 (0.81, 2.93)	1.03 (0.24, 4.34)	**3.23 (1.31, 7.77)**	**3.02 (1.28, 7.48)**	2.31 (0.81, 6.81)	1.11 (0.37, 3.49)	**2.44 (1.06, 5.76)**	IRd															
0.7 (0.12, 4.05)	0.41 (0.09, 1.53)	0.27 (0.04, 1.6)	0.86 (0.18, 3.49)	0.81 (0.18, 3.25)	0.62 (0.11, 2.81)	0.29 (0.05, 1.48)	0.65 (0.14, 2.51)	0.27 (0.06, 0.99)	Isa_KRd														
4.18 (0.86, 24.95)	2.5 (0.71, 8.71)	1.65 (0.3, 9.07)	**5.2 (1.34, 20.09)**	**4.88 (1.43, 17.64)**	3.74 (0.84, 16.71)	1.79 (0.38, 8.32)	**3.9 (1.17, 13.99)**	1.61 (0.47, 5.68)	**6.13 (1.18, 34.93)**	Isa_Rd													
1.49 (0.43, 6.35)	0.89 (0.41, 1.9)	0.59 (0.15, 2.35)	1.87 (0.72, 4.66)	1.75 (0.79, 3.9)	1.34 (0.43, 4.04)	0.64 (0.19, 2.16)	1.41 (0.67, 3)	0.58 (0.26, 1.23)	2.17 (0.58, 9.48)	**0.36 (0.13, 0.93)**	Isa_VRd												
2.11 (0.68, 8.17)	1.27 (0.63, 2.46)	0.85 (0.21, 3.35)	**2.64 (1.15, 5.93)**	**2.49 (1.17, 5.3)**	1.9 (0.68, 5.14)	0.91 (0.29, 2.86)	2 (0.97, 4.06)	0.82 (0.4, 1.58)	3.07 (0.96, 11.72)	0.51 (0.16, 1.58)	1.42 (0.75, 2.65)	KRd											
3.8 (0.7, 24.21)	2.21 (0.53, 9.91)	1.47 (0.22, 9.95)	**4.58 (1.04, 21.9)**	4.37 (0.99, 20.59)	3.32 (0.65, 18.12)	1.58 (0.29, 9.22)	3.52 (0.79, 16.11)	1.44 (0.34, 6.45)	5.5 (0.96, 36.8)	0.89 (0.16, 5.23)	2.49 (0.59, 11.02)	1.75 (0.49, 6.78)	KTd										
**4.58 (1.45, 18.41)**	**2.73 (1.75, 4.33)**	1.81 (0.46, 7.19)	**5.72 (2.64, 12.12)**	**5.37 (2.57, 11.63)**	**4.11 (1.57, 10.86)**	1.96 (0.72, 5.59)	**4.34 (2.12, 8.95)**	**1.77 (1.12, 2.82)**	**6.61 (1.92, 28.66)**	1.1 (0.34, 3.48)	**3.07 (1.66, 5.78)**	**2.15 (1.32, 3.67)**	1.23 (0.3, 4.8)	Rd									
**5.02 (1.43, 21.83)**	**2.97 (1.18, 7.54)**	1.97 (0.41, 9.59)	**6.18 (3.08, 12.77)**	**5.83 (2.02, 17.45)**	**4.45 (2.27, 8.99)**	2.14 (0.58, 7.87)	**4.71 (1.67, 13.76)**	1.93 (0.76, 4.94)	**7.26 (1.75, 35.81)**	1.19 (0.29, 4.8)	**3.34 (1.26, 9.17)**	**2.35 (1, 5.73)**	1.35 (0.27, 6.2)	1.08 (0.48, 2.46)	Td								
**7.43 (1.68, 38.09)**	**4.38 (1.47, 12.9)**	**2.86 (1.11, 7.97)**	**9.09 (2.75, 30.23)**	**8.55 (2.85, 26.63)**	**6.53 (1.7, 25.48)**	3.15 (0.75, 13.18)	**6.87 (2.42, 20.48)**	2.83 (0.97, 8.39)	**10.71 (2.36, 55.91)**	1.75 (0.43, 7.15)	**4.88 (1.82, 13.82)**	**3.44 (1.3, 9.41)**	1.96 (0.37, 10.22)	1.59 (0.6, 4.32)	1.47 (0.42, 5.11)	VCd							
**4.31 (1.26, 18.12)**	**2.58 (1.04, 6.27)**	1.69 (0.36, 8.03)	**5.37 (2.76, 10.33)**	**5.05 (1.79, 14.5)**	**3.85 (1.73, 8.5)**	1.85 (0.52, 6.6)	**4.05 (1.49, 11.32)**	1.67 (0.67, 4.06)	**6.21 (1.54, 30.5)**	1.03 (0.26, 4.07)	**2.87 (1.13, 7.59)**	2.03 (0.88, 4.73)	1.17 (0.24, 5.16)	0.94 (0.43, 2.04)	0.86 (0.45, 1.63)	0.59 (0.17, 1.96)	Vd						
2.51 (0.82, 9.65)	1.5 (0.7, 3.18)	0.99 (0.23, 4.29)	**3.12 (2, 4.87)**	**2.95 (1.18, 7.47)**	**2.25 (1.05, 4.85)**	1.07 (0.34, 3.54)	2.37 (0.98, 5.87)	0.97 (0.45, 2.07)	3.63 (0.97, 16.51)	0.6 (0.17, 2.12)	1.68 (0.75, 3.87)	1.19 (0.6, 2.37)	0.68 (0.15, 2.77)	0.55 (0.3, 1.01)	**0.51 (0.28, 0.88)**	0.34 (0.11, 1.03)	**0.58 (0.36, 0.95)**	VMP					
2.38 (0.75, 9.48)	1.43 (0.77, 2.61)	0.95 (0.25, 3.51)	**2.97 (1.32, 6.65)**	**2.78 (1.5, 5.37)**	2.14 (0.78, 5.9)	1.02 (0.35, 3.13)	**2.25 (1.27, 4.09)**	0.92 (0.5, 1.68)	**3.44 (1.02, 14.14)**	0.57 (0.19, 1.67)	**1.6 (1, 2.6)**	1.12 (0.76, 1.75)	0.64 (0.16, 2.46)	**0.52 (0.35, 0.78)**	0.48 (0.2, 1.14)	**0.33 (0.13, 0.79)**	0.55 (0.24, 1.26)	0.95 (0.49, 1.85)	VRd				
**6.2 (1.03, 42.32)**	3.64 (0.82, 16.1)	2.4 (0.46, 11.99)	**7.48 (1.54, 36.36)**	**7.12 (1.6, 32.61)**	**5.4 (1, 28.91)**	2.62 (0.46, 14.95)	**5.73 (1.32, 25.44)**	2.36 (0.53, 10.32)	**8.86 (1.36, 60.58)**	1.44 (0.25, 8.53)	4.02 (0.98, 17.78)	2.84 (0.7, 11.98)	1.63 (0.23, 10.67)	1.33 (0.32, 5.48)	1.21 (0.24, 5.88)	0.82 (0.23, 3.02)	1.41 (0.28, 6.86)	2.4 (0.52, 10.82)	2.53 (0.67, 10.03)	VRdC			
1.63 (0.46, 7.01)	0.97 (0.38, 2.44)	0.64 (0.13, 3.08)	**2.02 (1, 4.05)**	1.9 (0.66, 5.59)	1.45 (0.85, 2.5)	0.7 (0.19, 2.56)	1.53 (0.54, 4.39)	0.63 (0.25, 1.58)	2.36 (0.57, 11.6)	0.39 (0.09, 1.55)	1.08 (0.41, 2.92)	0.76 (0.32, 1.83)	0.44 (0.09, 2.02)	**0.35 (0.16, 0.79)**	**0.33 (0.21, 0.49)**	**0.22 (0.06, 0.77)**	**0.38 (0.21, 0.67)**	0.65 (0.37, 1.11)	0.68 (0.28, 1.6)	0.27 (0.05, 1.35)	VTd		
0.99 (0.14, 7.35)	0.59 (0.1, 3.06)	0.38 (0.04, 3.12)	1.21 (0.24, 5.76)	1.16 (0.19, 6.66)	0.88 (0.18, 3.85)	0.42 (0.06, 2.76)	0.93 (0.16, 5.15)	0.38 (0.07, 1.99)	1.44 (0.18, 11.61)	0.24 (0.03, 1.67)	0.65 (0.11, 3.6)	0.46 (0.08, 2.36)	0.26 (0.03, 2.07)	0.21 (0.04, 1.05)	**0.2 (0.04, 0.83)**	**0.13 (0.02, 0.86)**	0.23 (0.05, 1.02)	0.39 (0.08, 1.72)	0.41 (0.07, 2.06)	0.16 (0.02, 1.35)	0.6 (0.14, 2.39)	VTdC	
1.8 (0.53, 7.42)	1.07 (0.43, 2.57)	0.71 (0.15, 3.31)	**2.23 (1.16, 4.21)**	2.1 (0.75, 5.95)	1.59 (0.68, 3.79)	0.77 (0.22, 2.76)	1.68 (0.62, 4.69)	0.69 (0.28, 1.68)	2.58 (0.64, 12.44)	0.43 (0.11, 1.66)	1.2 (0.47, 3.12)	0.84 (0.37, 1.95)	0.48 (0.1, 2.15)	**0.39 (0.18, 0.83)**	**0.36 (0.18, 0.71)**	**0.24 (0.07, 0.81)**	**0.41 (0.24, 0.71)**	0.71 (0.44, 1.12)	0.75 (0.33, 1.7)	0.3 (0.06, 1.46)	1.1 (0.56, 2.14)	1.81 (0.39, 9.15)	VTP

Figure S5. League Plot for the NMA of ORR.

League table presenting ORs with 95% CIs for all pairwise comparisons. Each cell compares the column-defining intervention against the row-defining intervention. An OR with a 95% CI not i ncluding 1 indicates a statistically significant difference ($P < 0.05$). An OR > 1 favors the column -defining intervention. Treatment regimen abbreviations are defined in the List of Abbreviations.

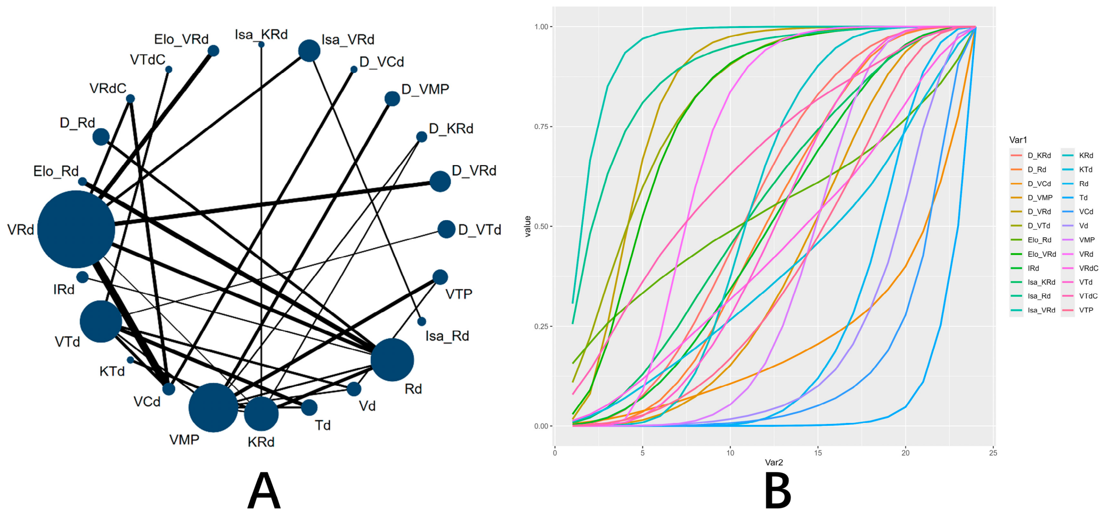

Figure S6. NMA of CR.

(A) Network plot of treatment comparisons. The size of each node is proportional to the number of participants assigned to that regimen, and the width of each line (edge) is proportional to the number of trials directly comparing the connected regimens. (B) SUCRA plot. A higher SUCRA value (closer to 100%) indicates a greater probability of being the most effective regimen for improving CR. Treatment regimen abbreviations are defined in the List of Abbreviations.

D_KRd																							
1.09 (0.48, 2.51)	D_Rd																						
2.43 (0.51, 12.64)	2.21 (0.47, 11.26)	D_VCd																					
1.27 (0.6, 2.68)	1.17 (0.51, 2.61)	0.52 (0.1, 2.52)	D_VMP																				
0.6 (0.28, 1.31)	0.55 (0.28, 1.09)	0.25 (0.05, 1.12)	0.48 (0.21, 1.05)	D_VRd																			
0.59 (0.23, 1.52)	0.55 (0.2, 1.45)	0.25 (0.05, 1.19)	0.47 (0.2, 1.09)	0.99 (0.38, 2.49)	D_VTd																		
0.93 (0.1, 8.58)	0.86 (0.09, 7.03)	0.38 (0.03, 5.02)	0.73 (0.07, 6.63)	1.54 (0.17, 13.15)	1.58 (0.15, 14.53)	Elo_Rd																	
0.66 (0.28, 1.58)	0.6 (0.27, 1.33)	0.27 (0.05, 1.29)	0.51 (0.22, 1.26)	1.1 (0.59, 2.01)	1.11 (0.4, 3.08)	0.7 (0.08, 6.81)	Elo_VRd																
1.1 (0.43, 2.83)	1.01 (0.5, 2.01)	0.45 (0.08, 2.3)	0.86 (0.35, 2.18)	1.83 (0.81, 4.06)	1.84 (0.64, 5.44)	1.17 (0.13, 11.19)	1.67 (0.68, 4.11)	IRd															
1.02 (0.41, 2.56)	0.94 (0.35, 2.48)	0.42 (0.07, 2.18)	0.8 (0.3, 2.13)	1.7 (0.69, 4.01)	1.72 (0.56, 5.4)	1.09 (0.11, 11.57)	1.56 (0.58, 4.02)	0.93 (0.32, 2.64)	Isa_KRd														
0.46 (0.15, 1.37)	0.42 (0.15, 1.17)	0.19 (0.03, 1.05)	0.36 (0.12, 1.1)	0.77 (0.31, 1.92)	0.78 (0.23, 2.64)	0.5 (0.05, 5.07)	0.7 (0.25, 1.89)	0.42 (0.14, 1.29)	0.46 (0.14, 1.48)	Isa_Rd													
0.41 (0.19, 0.93)	0.38 (0.18, 0.78)	0.17 (0.03, 0.79)	0.32 (0.14, 0.75)	0.68 (0.4, 1.17)	0.69 (0.26, 1.88)	0.44 (0.05, 4.24)	0.63 (0.32, 1.22)	0.37 (0.16, 0.87)	0.4 (0.16, 1.03)	0.89 (0.43, 1.86)	Isa_VRd												
1.01 (0.56, 1.84)	0.93 (0.48, 1.79)	0.42 (0.09, 1.87)	0.8 (0.4, 1.56)	**1.68 (1, 2.81)**	1.7 (0.73, 4.1)	1.09 (0.13, 10.18)	1.54 (0.79, 2.93)	0.92 (0.42, 2.03)	0.99 (0.49, 2.01)	2.19 (0.87, 5.55)	2.46 (1.37, 4.4)	KRd											
1.39 (0.4, 4.94)	1.28 (0.36, 4.69)	0.58 (0.09, 3.71)	1.11 (0.29, 4.1)	2.32 (0.69, 7.76)	2.35 (0.57, 10.04)	1.48 (0.13, 17.45)	2.12 (0.58, 7.68)	1.26 (0.33, 4.86)	1.36 (0.37, 5.11)	3 (0.69, 13.33)	3.39 (0.96, 11.65)	1.37 (0.46, 4.3)	KTd										
1.69 (0.82, 3.5)	**1.54 (1.04, 2.31)**	0.69 (0.14, 3.12)	1.32 (0.65, 2.72)	**2.8 (1.62, 4.85)**	**2.82 (1.18, 7.03)**	1.8 (0.23, 15.91)	**2.56 (1.28, 5.08)**	1.52 (0.86, 2.77)	1.65 (0.69, 4.02)	3.64 (1.41, 9.61)	4.08 (2.23, 7.49)	1.66 (0.99, 2.81)	1.2 (0.36, 4.06)	Rd									
3.55 (1.47, 8.66)	**3.25 (1.27, 8.2)**	1.46 (0.28, 6.88)	**2.79 (1.25, 6.11)**	**5.91 (2.38, 14.55)**	**5.98 (2.81, 12.74)**	3.83 (0.41, 40.97)	**5.4 (2.04, 14.29)**	**3.22 (1.15, 9.05)**	**3.49 (1.16, 10.19)**	**7.68 (2.29, 25.04)**	**8.66 (3.35, 21.72)**	**3.51 (1.56, 7.92)**	2.57 (0.64, 9.98)	2.12 (0.9, 4.91)	Td								
2.54 (0.91, 7.28)	2.3 (0.85, 6.69)	1.03 (0.31, 3.44)	1.98 (0.73, 5.6)	**4.14 (1.71, 10.87)**	4.23 (1.52, 12.53)	2.71 (0.27, 28.34)	**3.81 (1.42, 10.65)**	2.28 (0.77, 6.99)	2.45 (0.79, 7.9)	**5.44 (1.63, 18.52)**	**6.06 (2.4, 16.35)**	**2.46 (1.03, 6.36)**	1.81 (0.43, 7.47)	1.49 (0.6, 3.97)	0.71 (0.26, 2.05)	VCd							
1.86 (0.81, 4.34)	1.7 (0.7, 4.27)	0.77 (0.14, 3.73)	1.48 (0.71, 3.06)	**3.1 (1.3, 7.36)**	3.15 (1.37, 7.35)	2.01 (0.21, 20.88)	**2.83 (1.07, 7.43)**	1.69 (0.63, 4.7)	1.84 (0.63, 5.29)	**4.04 (1.27, 12.98)**	**4.51 (1.84, 11.24)**	1.84 (0.86, 4.02)	1.33 (0.36, 5.37)	1.1 (0.5, 2.51)	0.53 (0.23, 1.18)	0.75 (0.25, 2.08)	Vd						
1.28 (0.69, 2.33)	1.16 (0.58, 2.33)	0.53 (0.11, 2.33)	1 (0.65, 1.56)	**2.12 (1.09, 4.08)**	2.14 (1.05, 4.45)	1.37 (0.16, 13.13)	1.94 (0.89, 4.08)	1.16 (0.51, 2.63)	1.25 (0.52, 2.99)	**2.76 (1, 7.64)**	**3.09 (1.51, 6.2)**	1.26 (0.75, 2.11)	0.91 (0.27, 3.14)	0.76 (0.43, 1.34)	**0.36 (0.19, 0.7)**	0.51 (0.2, 1.22)	0.68 (0.38, 1.22)	VMP					
0.81 (0.41, 1.61)	0.74 (0.41, 1.34)	0.33 (0.07, 1.44)	0.64 (0.31, 1.29)	1.34 (0.96, 1.86)	1.35 (0.57, 3.31)	0.87 (0.11, 7.97)	1.22 (0.73, 2.04)	0.73 (0.35, 1.53)	0.79 (0.35, 1.82)	1.74 (0.75, 4.16)	**1.95 (1.28, 3)**	0.8 (0.54, 1.2)	0.58 (0.18, 1.86)	**0.48 (0.31, 0.75)**	**0.23 (0.1, 0.52)**	**0.32 (0.13, 0.73)**	**0.43 (0.19, 0.96)**	0.63 (0.36, 1.12)	VRd				
1.24 (0.35, 4.74)	1.13 (0.33, 4.11)	0.51 (0.09, 2.98)	0.98 (0.27, 3.66)	2.05 (0.66, 6.7)	2.1 (0.54, 8.31)	1.35 (0.12, 15.63)	1.87 (0.55, 6.54)	1.13 (0.31, 4.31)	1.21 (0.32, 4.92)	2.68 (0.67, 11.18)	2.99 (0.95, 10.16)	1.22 (0.39, 4.01)	0.89 (0.18, 4.58)	0.73 (0.23, 2.51)	0.35 (0.09, 1.38)	0.5 (0.14, 1.77)	0.66 (0.18, 2.59)	0.97 (0.3, 3.42)	1.54 (0.52, 4.78)	VRdC			
1.11 (0.51, 2.37)	1.02 (0.44, 2.25)	0.46 (0.09, 2.04)	0.88 (0.45, 1.69)	1.85 (0.84, 3.94)	1.87 (1.1, 3.19)	1.19 (0.13, 11.78)	1.69 (0.7, 4.01)	1.01 (0.39, 2.54)	1.1 (0.4, 2.94)	2.42 (0.8, 7.12)	**2.7 (1.18, 6)**	1.1 (0.55, 2.16)	0.8 (0.21, 2.97)	0.66 (0.32, 1.33)	**0.31 (0.18, 0.54)**	0.44 (0.17, 1.06)	0.6 (0.31, 1.13)	0.87 (0.54, 1.42)	1.38 (0.67, 2.74)	0.9 (0.25, 3.09)	VTd		
0.78 (0.22, 2.72)	0.72 (0.19, 2.59)	0.32 (0.05, 1.94)	0.62 (0.18, 2.05)	1.31 (0.36, 4.73)	1.31 (0.42, 4.12)	0.84 (0.08, 10.77)	1.2 (0.3, 4.54)	0.71 (0.18, 2.79)	0.77 (0.19, 3.14)	1.7 (0.37, 7.55)	1.9 (0.5, 7.18)	0.77 (0.22, 2.64)	0.56 (0.1, 3.01)	0.47 (0.13, 1.6)	**0.22 (0.07, 0.69)**	0.31 (0.08, 1.19)	0.42 (0.13, 1.36)	0.62 (0.2, 1.89)	0.97 (0.28, 3.37)	0.64 (0.12, 3.06)	0.71 (0.26, 1.98)	VTdC	
1.32 (0.56, 3.12)	1.2 (0.49, 3.04)	0.55 (0.1, 2.67)	1.04 (0.49, 2.2)	2.19 (0.9, 5.33)	2.22 (0.92, 5.58)	1.42 (0.15, 14.57)	2.01 (0.74, 5.3)	1.2 (0.43, 3.34)	1.3 (0.44, 3.73)	2.86 (0.86, 9.13)	**3.21 (1.24, 8.01)**	1.31 (0.58, 2.89)	0.94 (0.24, 3.78)	0.78 (0.34, 1.81)	**0.37 (0.15, 0.9)**	0.53 (0.18, 1.5)	0.71 (0.37, 1.37)	1.03 (0.56, 1.91)	1.64 (0.71, 3.71)	1.06 (0.27, 4.04)	1.18 (0.58, 2.49)	1.68 (0.47, 5.8)	VTP

Figure S7. League Plot for the NMA of CR.

League table presenting ORs with 95% CIs for all pairwise comparisons. Each cell compares the column-defining intervention against the row-defining intervention. An OR with a 95% CI not including 1 indicates a statistically significant difference ($P < 0.05$). An $OR > 1$ favors the column -defining intervention. Treatment regimen abbreviations are defined in the List of Abbreviations.

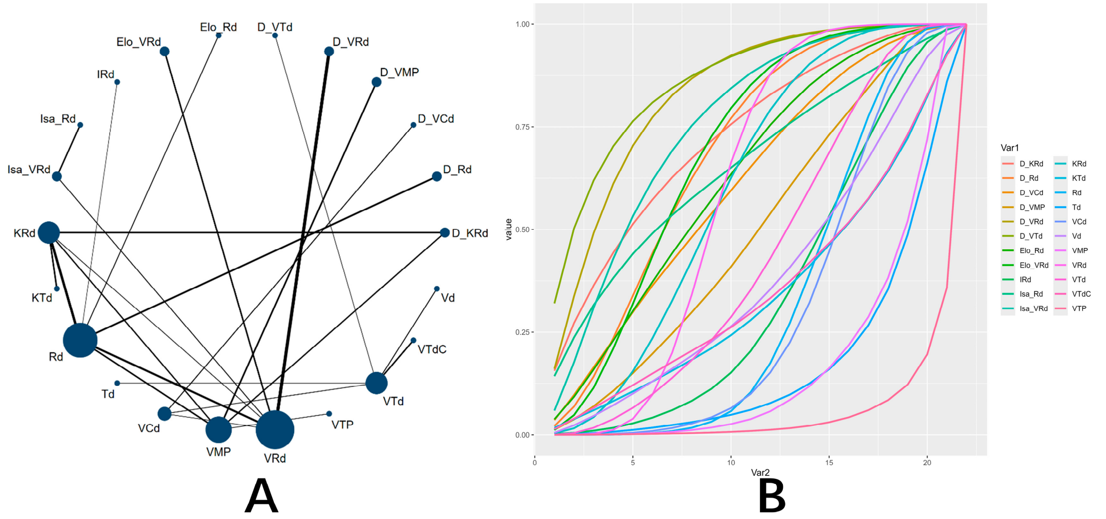

Figure S8. NMA of OS.

(A) Network plot of treatment comparisons. The size of each node is proportional to the number of participants assigned to that regimen, and the width of each line (edge) is proportional to the number of trials directly comparing the connected regimens. (B) SUCRA plot. A higher SUCRA value (closer to 100%) indicates a greater probability of being the most effective regimen for improving OS. Treatment regimen abbreviations are defined in the List of Abbreviations.

D_KRd																					
0.9 (0.38, 2.13)	D_Rd																				
0.84 (0.29, 2.44)	0.93 (0.4, 2.22)	D_VCd																			
0.72 (0.34, 1.54)	0.8 (0.38, 1.71)	0.86 (0.32, 2.34)	D_VMP																		
1.13 (0.44, 2.86)	1.25 (0.64, 2.47)	1.34 (0.57, 3.15)	1.57 (0.67, 3.63)	D_VRd																	
1.23 (0.4, 3.69)	1.36 (0.55, 3.43)	1.46 (0.62, 3.42)	1.71 (0.6, 4.78)	1.09 (0.44, 2.73)	D_VTd																
0.86 (0.33, 2.23)	0.95 (0.51, 1.79)	1.02 (0.4, 2.63)	1.19 (0.5, 2.79)	0.76 (0.34, 1.68)	0.7 (0.26, 1.9)	Elo_Rd															
0.91 (0.38, 2.13)	1 (0.56, 1.82)	1.07 (0.49, 2.36)	1.25 (0.57, 2.7)	0.8 (0.45, 1.46)	0.73 (0.31, 1.73)	1.05 (0.51, 2.17)	Elo_VRd														
0.62 (0.25, 1.52)	0.69 (0.41, 1.2)	0.73 (0.31, 1.81)	0.86 (0.39, 1.89)	0.55 (0.27, 1.14)	0.5 (0.2, 1.32)	0.73 (0.37, 1.43)	0.69 (0.36, 1.31)	IRd													
0.93 (0.28, 3.17)	1.04 (0.36, 3)	1.11 (0.35, 3.62)	1.3 (0.4, 4.13)	0.83 (0.29, 2.37)	0.76 (0.23, 2.55)	1.1 (0.35, 3.42)	1.04 (0.38, 2.78)	1.52 (0.51, 4.4)	Isa_Rd												
1.01 (0.39, 2.62)	1.12 (0.55, 2.3)	1.2 (0.49, 2.91)	1.4 (0.58, 3.34)	0.89 (0.44, 1.84)	0.82 (0.32, 2.11)	1.18 (0.52, 2.71)	1.12 (0.59, 2.1)	1.63 (0.76, 3.43)	1.08 (0.5, 2.33)	Isa_VRd											
0.82 (0.4, 1.72)	0.91 (0.51, 1.66)	0.97 (0.43, 2.25)	1.14 (0.59, 2.2)	0.73 (0.39, 1.39)	0.67 (0.28, 1.64)	0.96 (0.48, 1.97)	0.91 (0.54, 1.57)	1.33 (0.71, 2.49)	0.88 (0.32, 2.45)	0.81 (0.42, 1.61)	KRd										
0.59 (0.2, 1.75)	0.65 (0.25, 1.78)	0.69 (0.23, 2.23)	0.81 (0.29, 2.28)	0.52 (0.19, 1.45)	0.48 (0.15, 1.58)	0.69 (0.24, 2.01)	0.65 (0.26, 1.71)	0.94 (0.35, 2.59)	0.62 (0.17, 2.31)	0.58 (0.21, 1.65)	0.71 (0.33, 1.6)	KTd									
0.61 (0.28, 1.36)	**0.68 (0.49, 0.97)**	0.73 (0.33, 1.62)	0.85 (0.43, 1.68)	**0.54 (0.3, 0.99)**	0.5 (0.21, 1.18)	0.72 (0.42, 1.22)	0.68 (0.42, 1.11)	0.99 (0.65, 1.5)	0.65 (0.24, 1.79)	0.61 (0.32, 1.15)	0.75 (0.46, 1.2)	1.05 (0.41, 2.61)	Rd								
0.46 (0.15, 1.38)	0.51 (0.2, 1.28)	0.54 (0.23, 1.27)	0.63 (0.22, 1.77)	0.4 (0.16, 1.02)	**0.37 (0.2, 0.69)**	0.53 (0.2, 1.46)	0.51 (0.22, 1.18)	0.74 (0.28, 1.88)	0.48 (0.15, 1.65)	0.45 (0.18, 1.16)	0.55 (0.23, 1.33)	0.78 (0.24, 2.48)	0.75 (0.32, 1.72)	Td							
0.6 (0.24, 1.48)	0.66 (0.35, 1.28)	0.71 (0.41, 1.24)	0.83 (0.36, 1.88)	0.53 (0.28, 1.02)	**0.49 (0.26, 0.93)**	0.7 (0.33, 1.52)	0.67 (0.38, 1.15)	0.97 (0.48, 1.92)	0.64 (0.23, 1.79)	0.59 (0.3, 1.18)	0.73 (0.4, 1.31)	1.03 (0.38, 2.69)	0.98 (0.56, 1.69)	1.31 (0.69, 2.49)	VCd						
0.62 (0.2, 1.96)	0.69 (0.26, 1.83)	0.74 (0.3, 1.84)	0.87 (0.29, 2.54)	0.55 (0.21, 1.46)	0.51 (0.25, 1.02)	0.73 (0.25, 2.08)	0.69 (0.28, 1.7)	1.01 (0.37, 2.71)	0.66 (0.19, 2.34)	0.62 (0.23, 1.67)	0.76 (0.29, 1.91)	1.07 (0.31, 3.54)	1.02 (0.41, 2.5)	1.37 (0.68, 2.74)	1.04 (0.51, 2.13)	Vd					
**0.46 (0.23, 0.91)**	0.51 (0.26, 1.01)	0.55 (0.22, 1.4)	**0.64 (0.45, 0.89)**	**0.41 (0.19, 0.89)**	0.37 (0.14, 1.01)	0.54 (0.24, 1.19)	0.51 (0.25, 1.02)	0.74 (0.36, 1.51)	0.49 (0.16, 1.49)	0.46 (0.2, 1.02)	**0.56 (0.32, 0.97)**	0.78 (0.3, 2.07)	0.75 (0.41, 1.34)	1.01 (0.38, 2.7)	0.77 (0.36, 1.62)	0.74 (0.26, 2.09)	VMP				
0.82 (0.37, 1.8)	0.91 (0.56, 1.48)	0.97 (0.48, 1.97)	1.13 (0.57, 2.26)	0.72 (0.45, 1.17)	0.66 (0.31, 1.45)	0.95 (0.51, 1.8)	0.9 (0.64, 1.28)	1.32 (0.76, 2.24)	0.87 (0.34, 2.23)	0.81 (0.48, 1.37)	0.99 (0.65, 1.49)	1.4 (0.57, 3.32)	1.33 (0.94, 1.87)	1.78 (0.82, 3.89)	1.36 (0.88, 2.1)	1.31 (0.57, 3.03)	1.77 (0.97, 3.26)	VRd			
0.68 (0.24, 1.86)	0.75 (0.34, 1.68)	0.8 (0.39, 1.67)	0.94 (0.36, 2.38)	0.6 (0.27, 1.34)	**0.55 (0.36, 0.85)**	0.79 (0.32, 1.95)	0.75 (0.36, 1.55)	1.09 (0.47, 2.49)	0.72 (0.23, 2.24)	0.67 (0.29, 1.53)	0.82 (0.38, 1.74)	1.16 (0.38, 3.36)	1.11 (0.53, 2.25)	1.48 (0.96, 2.29)	1.13 (0.7, 1.81)	1.09 (0.63, 1.87)	1.46 (0.6, 3.52)	0.83 (0.43, 1.57)	VTd		
0.59 (0.16, 2.1)	0.65 (0.22, 1.96)	0.7 (0.24, 2.05)	0.82 (0.24, 2.73)	0.52 (0.17, 1.58)	0.48 (0.2, 1.15)	0.69 (0.21, 2.22)	0.65 (0.23, 1.85)	0.95 (0.31, 2.91)	0.62 (0.16, 2.45)	0.58 (0.19, 1.77)	0.72 (0.24, 2.07)	1 (0.26, 3.75)	0.96 (0.33, 2.71)	1.29 (0.54, 3.09)	0.98 (0.4, 2.39)	0.94 (0.37, 2.39)	1.28 (0.4, 4.09)	0.72 (0.27, 1.94)	0.87 (0.41, 1.86)	VTdC	
**0.31 (0.14, 0.7)**	**0.34 (0.15, 0.78)**	0.37 (0.13, 1.05)	**0.43 (0.24, 0.75)**	**0.27 (0.11, 0.67)**	**0.25 (0.09, 0.75)**	**0.36 (0.15, 0.9)**	**0.34 (0.15, 0.79)**	0.5 (0.21, 1.16)	0.33 (0.1, 1.1)	**0.31 (0.12, 0.77)**	**0.38 (0.18, 0.76)**	0.52 (0.18, 1.54)	0.5 (0.24, 1.05)	0.68 (0.23, 2)	0.52 (0.22, 1.24)	0.49 (0.16, 1.53)	0.67 (0.42, 1.06)	**0.38 (0.18, 0.81)**	0.46 (0.17, 1.24)	0.53 (0.15, 1.81)	VTP

Figure S9. League Plot for the NMA of OS.

League table presenting HRs with 95% CIs for all pairwise comparisons. Each cell compares the column-defining intervention against the row-defining intervention. An HR with a 95% CI not including 1 indicates a statistically significant difference ($P < 0.05$). An HR < 1 favors the column-defining intervention. Treatment regimen abbreviations are defined in the List of Abbreviations.

Study or Subgroup	Experimental Events	Experimental Total	Control Events	Control Total	Weight	Odds Ratio M-H, Fixed, 95% CI
**1.1.1 TEMM**						
CASSIOPEIA 2024	503	543	486	542	11.1%	1.45 [0.95, 2.22]
GIMEMA 2020	207	241	165	239	7.2%	2.73 [1.73, 4.30]
GMMG-HD7 2025	299	331	284	329	8.5%	1.48 [0.91, 2.40]
GRIFFIN 2023	103	104	95	103	0.3%	8.67 [1.06, 70.65]
Heinz 2015	45	49	43	49	1.1%	1.57 [0.41, 5.95]
IFM 2007-02 2011	87	100	72	99	2.9%	2.51 [1.21, 5.22]
IsKia 2023	147	151	140	151	1.1%	2.89 [0.90, 9.28]
Lalit 2019	58	65	45	60	1.6%	2.76 [1.04, 7.34]
PERSEUS 2024	343	355	332	354	3.5%	1.89 [0.92, 3.89]
**Subtotal (95% CI)**		**1939**		**1926**	**37.3%**	**1.99 [1.60, 2.46]**
Total events	1792		1662			
Heterogeneity: Chi² = 8.69, df = 8 (P = 0.37); I² = 8%						
Test for overall effect: Z = 6.23 (P < 0.00001)						
**1.1.2 TIEMM**						
AGMT-MM02 2024	56	61	53	61	1.3%	1.69 [0.52, 5.50]
ALCYONE 2025	324	356	259	350	7.3%	3.56 [2.30, 5.49]
AMaRC 2024	55	64	39	57	1.8%	2.82 [1.15, 6.93]
BENEFIT/IFM 2024	128	135	118	135	1.9%	2.63 [1.06, 6.58]
EMN20 2024	35	42	24	40	1.3%	3.33 [1.19, 9.33]
IFM2017-03 2022	177	200	73	95	3.5%	2.32 [1.22, 4.42]
IMROZ 2024	253	265	166	181	2.8%	1.91 [0.87, 4.17]
Kohmei 2020	31	40	27	42	1.8%	1.91 [0.72, 5.07]
MAIA 2025	342	368	301	369	6.6%	2.97 [1.84, 4.79]
Mookerjee 2017	61	74	51	69	2.9%	1.66 [0.74, 3.70]
OCTANS 2025	129	146	57	74	2.7%	2.26 [1.08, 4.75]
Saad 2025	194	197	187	198	0.9%	3.80 [1.04, 13.85]
SWOG-1211 2021	45	48	43	52	0.8%	3.14 [0.80, 12.38]
SWOG-S0777 2017	216	264	188	261	10.6%	1.75 [1.16, 2.64]
TOURMALINE 2020	269	351	230	354	16.6%	1.77 [1.27, 2.46]
**Subtotal (95% CI)**		**2611**		**2338**	**62.7%**	**2.29 [1.95, 2.69]**
Total events	2315		1816			
Heterogeneity: Chi² = 11.91, df = 14 (P = 0.61); I² = 0%						
Test for overall effect: Z = 10.13 (P < 0.00001)						
**Total (95% CI)**		**4550**		**4264**	**100.0%**	**2.18 [1.91, 2.47]**
Total events	4107		3478			
Heterogeneity: Chi² = 21.71, df = 23 (P = 0.54); I² = 0%						
Test for overall effect: Z = 11.85 (P < 0.00001)						
Test for subgroup differences: Chi² = 1.06, df = 1 (P = 0.30), I² = 5.8%						

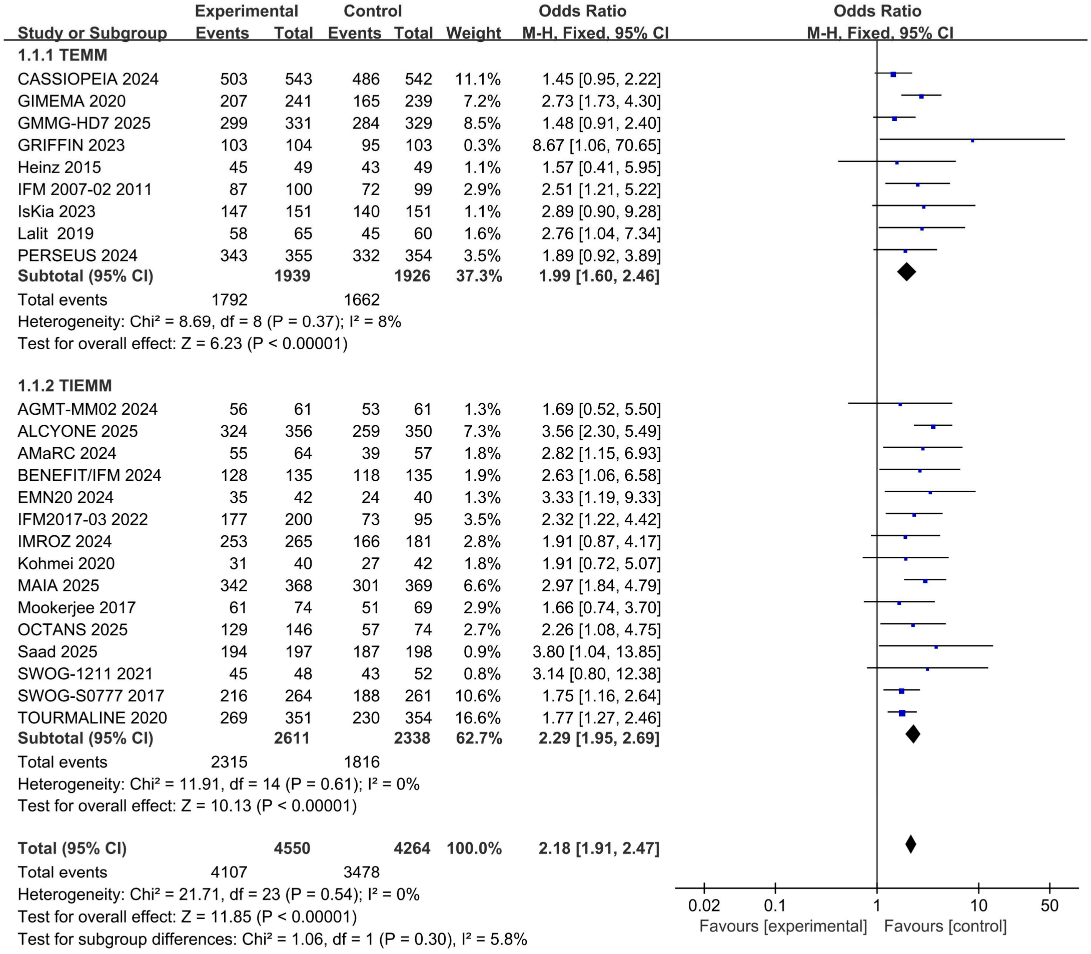

Figure S10. Subgroup Analysis of ORR in TE and TIE Populations.

Study or Subgroup	Experimental Events	Experimental Total	Control Events	Control Total	Weight	Odds Ratio M-H, Fixed, 95% CI
**1.4.1 TEMM**						
CASSIOPEIA 2024	55	543	31	542	7.4%	1.86 [1.18, 2.94]
GIMEMA 2020	39	241	10	239	2.2%	4.42 [2.15, 9.08]
GMMG-HD6 2021	40	279	34	280	7.7%	1.21 [0.74, 1.98]
GMMG-HD7 2025	52	331	26	329	5.8%	2.17 [1.32, 3.57]
GRIFFIN 2023	21	104	15	103	3.2%	1.48 [0.72, 3.07]
Heinz 2015	13	49	10	49	1.9%	1.41 [0.55, 3.61]
IFM 2007-02 2011	12	100	8	99	1.9%	1.55 [0.61, 3.98]
IFM2013-04	11	170	4	170	1.0%	2.87 [0.90, 9.20]
IsKia 2023	20	151	20	151	4.6%	1.00 [0.51, 1.95]
Lalit 2019	8	65	2	60	0.5%	4.07 [0.83, 20.00]
PERSEUS 2024	62	355	49	354	10.7%	1.32 [0.88, 1.98]
**Subtotal (95% CI)**		**2388**		**2376**	**46.7%**	**1.69 [1.41, 2.04]**
Total events	333		209			
Heterogeneity: Chi² = 15.86, df = 10 (P = 0.10); I² = 37%						
Test for overall effect: Z = 5.59 (P < 0.00001)						
**1.4.2 TIEMM**						
AGMT-MM02 2024	9	61	7	61	1.6%	1.34 [0.46, 3.85]
AMaRC 2024	7	64	6	57	1.5%	1.04 [0.33, 3.31]
BENEFIT/IFM 2024	22	135	20	135	4.4%	1.12 [0.58, 2.16]
EMN20 2024	7	42	4	40	0.9%	1.80 [0.48, 6.70]
GEM2005 2014	13	130	10	130	2.4%	1.33 [0.56, 3.16]
IFM2017-03 2022	27	200	10	95	3.1%	1.33 [0.61, 2.87]
IMROZ 2024	41	265	18	181	4.8%	1.66 [0.92, 2.99]
Kohmei 2020	3	40	2	42	0.5%	1.62 [0.26, 10.25]
MAIA 2025	86	368	59	369	11.9%	1.60 [1.11, 2.32]
OCTANS 2025	20	146	10	74	3.0%	1.02 [0.45, 2.30]
Saad 2025	40	197	33	198	6.9%	1.27 [0.76, 2.12]
SWOG-1211 2021	6	48	5	52	1.1%	1.34 [0.38, 4.72]
SWOG-S0777 2017	42	264	22	261	4.9%	2.06 [1.19, 3.55]
TOURMALINE 2020	39	351	27	354	6.3%	1.51 [0.90, 2.53]
**Subtotal (95% CI)**		**2311**		**2049**	**53.3%**	**1.47 [1.23, 1.76]**
Total events	362		233			
Heterogeneity: Chi² = 4.18, df = 13 (P = 0.99); I² = 0%						
Test for overall effect: Z = 4.22 (P < 0.0001)						
**Total (95% CI)**		**4699**		**4425**	**100.0%**	**1.57 [1.38, 1.79]**
Total events	695		442			
Heterogeneity: Chi² = 20.88, df = 24 (P = 0.65); I² = 0%						
Test for overall effect: Z = 6.92 (P < 0.00001)						
Test for subgroup differences: Chi² = 1.17, df = 1 (P = 0.28), I² = 14.4%						

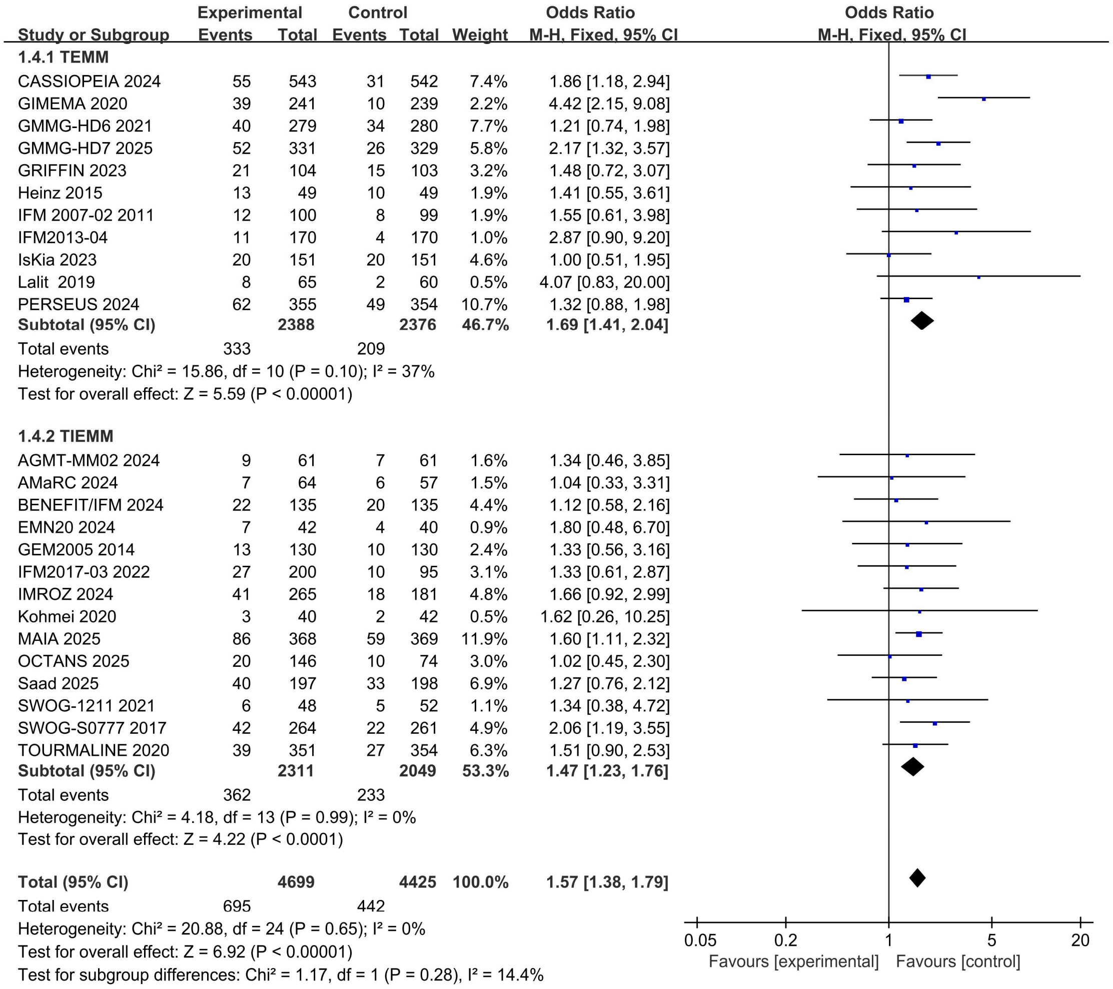

Figure S11. Subgroup Analysis of CR in TE and TIE Populations.

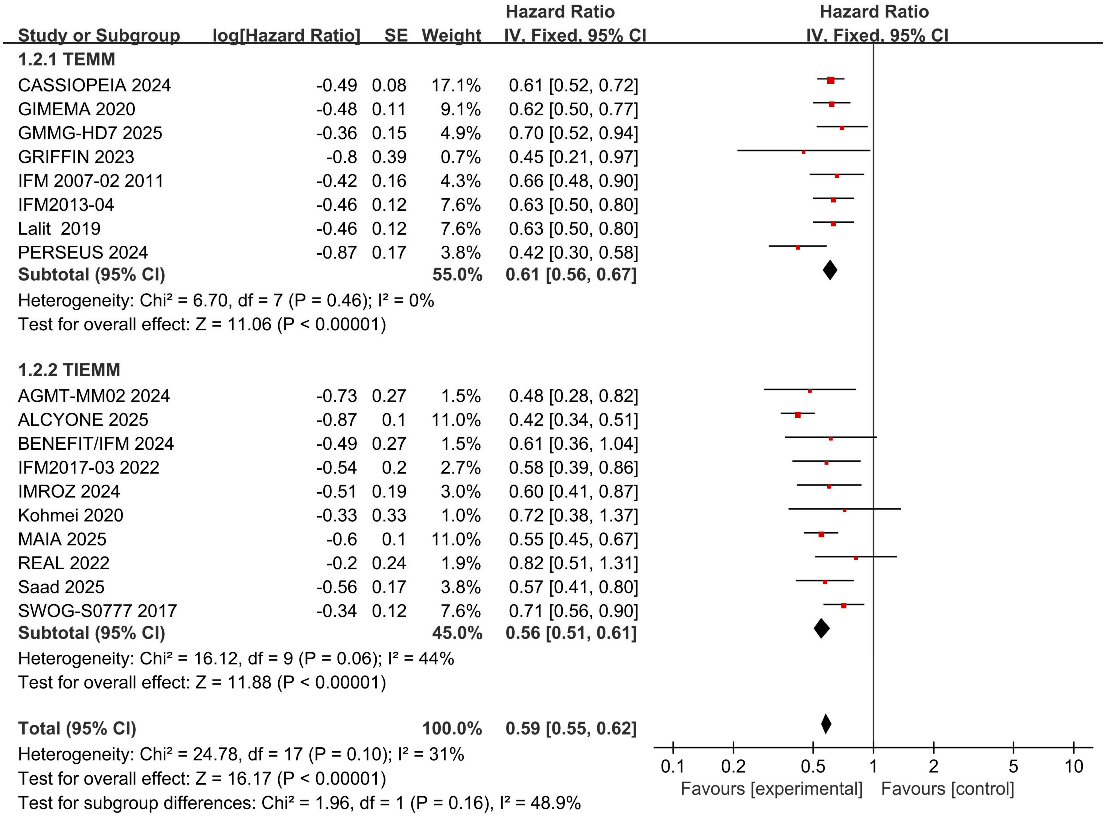

Figure S12. Subgroup Analysis of PFS in TE and TIE Populations.

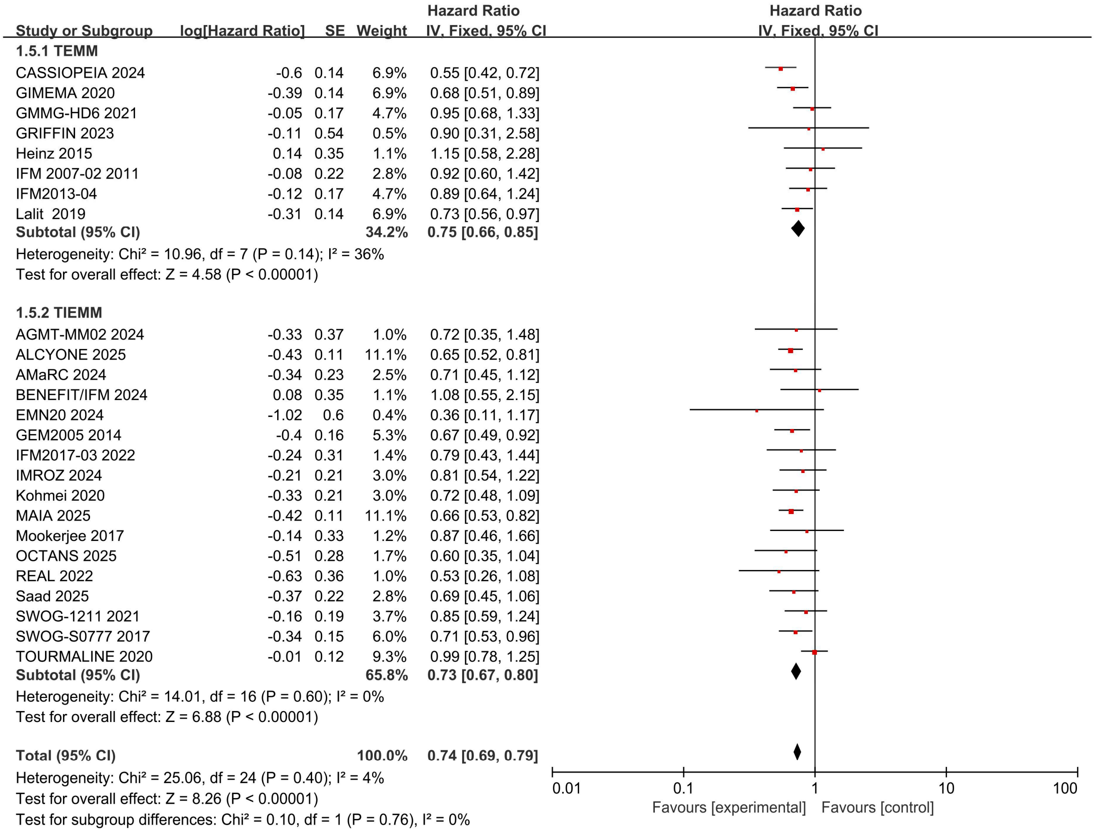

Figure S13. Subgroup Analysis of OS in TE and TIE Populations.

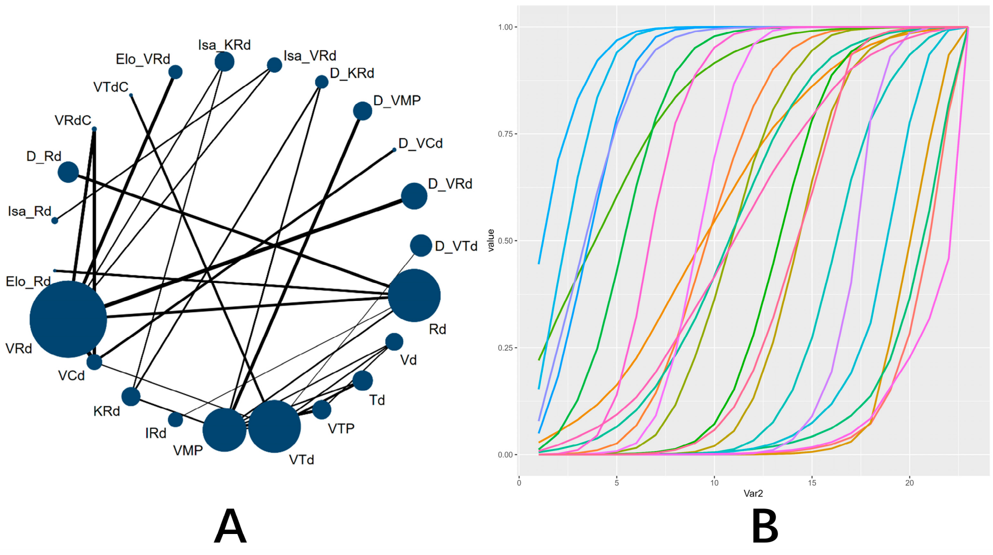

Figure S14. NMA of Grade ≥3AEs.

(A) Network plot of treatment comparisons. The size of each node is proportional to the number of participants assigned to that regimen, and the width of each line (edge) is proportional to the number of trials directly comparing the connected regimens. (B) SUCRA plot. A higher SUCRA value (closer to 100%) indicates that the regimen is associated with a lower risk of Grade ≥3 AEs. Treatment regimen abbreviations are defined in the List of Abbreviations.

D_KRd																						
**4.11 (1.49,11.41)**	D_Rd																					
**4.16 (1.08, 15.81)**	1.01 (0.29, 3.43)	D_VCd																				
1.16 (0.51, 2.63)	**0.28 (0.12, 0.64)**	**0.28 (0.08, 0.96)**	D_VMP																			
2.49 (0.93, 6.76)	0.6 (0.32, 1.17)	0.59 (0.19, 2.04)	2.14 (0.97, 4.94)	D_VRd																		
**3.55 (1.45, 9.13)**	0.87 (0.38, 1.98)	0.85 (0.27, 2.83)	**3.08 (1.56, 6.24)**	1.44 (0.65, 3.1)	D_VTd																	
**7.91 (2.06, 29.2)**	1.91 (0.66, 5.35)	1.88 (0.42, 8.16)	**6.82 (2.01,21.55)**	**3.15 (1.05, 8.99)**	2.22 (0.65, 6.98)	Elo_Rd																
**2.76 (1, 7.69)**	0.67 (0.34, 1.36)	0.65 (0.2, 2.34)	**2.37 (1.05, 5.55)**	1.11 (0.66, 1.88)	0.76 (0.35, 1.75)	**0.35 (0.12, 1.07)**	Elo_VRd															
**6.58 (2.43,17.92)**	1.6 (0.89, 2.85)	1.57 (0.47, 5.57)	**5.67 (2.56,13.15)**	**2.65 (1.41, 4.89)**	1.85 (0.82, 4.14)	0.83 (0.31, 2.41)	**2.4 (1.21, 4.74)**	IRd														
1.06 (0.42, 2.74)	**0.26 (0.08, 0.83)**	0.26 (0.06, 1.1)	0.91 (0.35, 2.46)	0.43 (0.13, 1.33)	**0.3 (0.1, 0.86)**	**0.13 (0.03, 0.57)**	0.39 (0.12, 1.22)	**0.16 (0.05, 0.5)**	Isa_KRd													
**3.58 (1.07,11.98)**	0.86 (0.34, 2.3)	0.85 (0.22, 3.58)	**3.06 (1.07, 9.04)**	1.43 (0.62, 3.32)	0.99 (0.36, 2.91)	0.45 (0.13, 1.67)	1.3 (0.54, 3.07)	0.54 (0.21, 1.41)	3.37 (0.9, 12.75)	Isa_Rd												
2 (0.71, 5.65)	**0.48 (0.24, 0.99)**	0.48 (0.15, 1.71)	1.73 (0.74, 4.2)	0.81 (0.46, 1.38)	0.56 (0.24, 1.34)	**0.25 (0.09, 0.78)**	0.73 (0.4, 1.31)	**0.3 (0.15, 0.61)**	1.89 (0.59, 6.24)	0.56 (0.29, 1.05)	Isa_VRd											
1.39 (0.66, 2.93)	**0.34 (0.12, 0.92)**	0.33 (0.09, 1.29)	1.19 (0.55, 2.61)	0.56 (0.21, 1.47)	**0.39 (0.16, 0.94)**	**0.18 (0.05, 0.66)**	0.5 (0.19, 1.36)	**0.21 (0.08, 0.55)**	1.3 (0.72, 2.4)	0.39 (0.12, 1.27)	0.69 (0.25, 1.91)	KRd										
**9.11 (3.65,23.15)**	2.21 (1.45, 3.43)	2.18 (0.7, 7.17)	**7.87 (3.92,16.43)**	**3.67 (2.25, 5.9)**	**2.56 (1.25, 5.25)**	1.16 (0.46, 3.1)	**3.32 (1.9, 5.7)**	1.39 (0.94, 2.04)	**8.61 (2.94, 25.8)**	**2.57 (1.08, 5.92)**	**4.55 (2.58, 8.01)**	**6.59 (2.73, 16.62)**	Rd									
**10.61 (4.54,26.09)**	2.58 (1.16, 5.72)	2.54 (0.83, 8.32)	**9.16 (4.94, 17.63)**	**4.28 (2.02, 8.99)**	**2.97 (1.83, 4.94)**	1.34 (0.44, 4.4)	**3.86 (1.74, 8.4)**	1.61 (0.74, 3.52)	**10.08 (3.61, 27.93)**	**3 (1.05, 8.26)**	**5.31 (2.32, 11.85)**	**7.69 (3.37, 18.02)**	1.16 (0.6, 2.27)	Td								
**8.04 (3.3, 20.29)**	1.96 (0.94, 4.04)	1.93 (0.72, 5.52)	**6.96 (3.52, 14.24)**	**3.27 (1.71, 5.96)**	**2.26 (1.26, 4.06)**	1.02 (0.35, 3.23)	**2.95 (1.49, 5.61)**	1.23 (0.6, 2.45)	**7.65 (2.67, 22.11)**	2.29 (0.85, 5.72)	**4.06 (1.97, 8.06)**	**5.79 (2.46, 14.41)**	0.89 (0.49, 1.57)	0.76 (0.43, 1.33)	VCd							
**8.23 (3.62, 19.61)**	2.01 (0.89, 4.5)	1.98 (0.61, 6.67)	**7.14 (3.99,13.14)**	**3.34 (1.52, 7.14)**	**2.31 (1.29, 4.18)**	1.04 (0.34, 3.48)	**3 (1.34, 6.66)**	1.26 (0.56, 2.75)	**7.8 (2.87, 21.44)**	2.32 (0.81, 6.55)	**4.14 (1.77, 9.34)**	**5.97 (2.69, 13.56)**	0.9 (0.45, 1.79)	0.78 (0.45, 1.33)	1.02 (0.55, 1.89)	Vd						
1.76 (0.87, 3.66)	**0.43 (0.21, 0.87)**	0.42 (0.14, 1.39)	**1.52 (1.03, 2.32)**	0.71 (0.35, 1.39)	**0.49 (0.28, 0.86)**	**0.22 (0.08, 0.7)**	0.64 (0.31, 1.31)	**0.27 (0.13, 0.53)**	1.67 (0.67, 4.12)	0.5 (0.18, 1.31)	0.88 (0.4, 1.85)	1.27 (0.65, 2.55)	**0.19 (0.11, 0.34)**	**0.17 (0.1, 0.27)**	**0.22 (0.12, 0.38)**	**0.21 (0.14, 0.33)**	VMP					
**4.26 (1.69,10.92)**	1.03 (0.6, 1.83)	1.01 (0.34, 3.35)	**3.67 (1.77, 7.83)**	**1.71 (1.24, 2.37)**	1.19 (0.59, 2.45)	0.54 (0.2, 1.54)	**1.55 (1.02, 2.33)**	0.64 (0.38, 1.1)	**4.02 (1.35, 12.14)**	1.19 (0.55, 2.58)	**2.11 (1.38, 3.31)**	**3.06 (1.24, 7.74)**	**0.46 (0.33, 0.67)**	**0.4 (0.21, 0.79)**	**0.52 (0.31, 0.91)**	0.51 (0.26, 1.05)	**2.4 (1.33, 4.51)**	VRd				
0.72 (0.16, 2.98)	**0.17 (0.04, 0.63)**	**0.17 (0.03, 0.81)**	0.63 (0.15, 2.27)	**0.29 (0.08, 0.95)**	**0.2 (0.05, 0.7)**	**0.09 (0.02, 0.43)**	**0.26 (0.07, 0.87)**	**0.11 (0.03, 0.38)**	0.68 (0.13, 3.09)	**0.2 (0.05, 0.81)**	0.36 (0.09, 1.22)	0.52 (0.11, 2.1)	**0.08 (0.02, 0.26)**	**0.07 (0.02, 0.23)**	**0.09 (0.02, 0.27)**	**0.09 (0.02, 0.31)**	0.41 (0.11, 1.39)	**0.17 (0.05, 0.52)**	VRdC			
**5.59 (2.48, 13.3)**	1.36 (0.64, 2.88)	1.34 (0.45, 4.18)	**4.84 (2.71, 8.84)**	**2.26 (1.11, 4.44)**	**1.57 (1.09, 2.26)**	0.71 (0.24, 2.28)	2.04 (0.98, 4.18)	0.85 (0.41, 1.76)	**5.27 (1.94, 14.44)**	1.59 (0.57, 4.15)	**2.81 (1.29, 5.95)**	**4.05 (1.83, 9.19)**	0.61 (0.33, 1.13)	**0.53 (0.37, 0.73)**	0.69 (0.44, 1.1)	0.68 (0.42, 1.08)	**3.18 (2.06, 4.95)**	1.32 (0.71, 2.4)	**7.69 (2.39, 30.26)**	VTd		
3.53 (0.94,12.87)	0.86 (0.24, 2.88)	0.85 (0.2, 3.61)	3.06 (0.93, 9.33)	1.43 (0.42, 4.53)	0.99 (0.33, 2.73)	0.45 (0.1, 2.07)	1.28 (0.37, 4.23)	0.54 (0.15, 1.78)	3.34 (0.79, 13.16)	0.99 (0.24, 3.89)	1.76 (0.5, 5.94)	2.56 (0.69, 8.87)	0.39 (0.12, 1.2)	**0.33 (0.11, 0.9)**	0.44 (0.14, 1.24)	0.43 (0.14, 1.22)	2.01 (0.67, 5.68)	0.83 (0.26, 2.56)	**4.89 (1.02, 25.96)**	0.63 (0.23, 1.62)	VTdC	
**2.51 (1.09, 5.93)**	0.61 (0.26, 1.38)	0.6 (0.18, 2.08)	**2.17 (1.22, 3.87)**	1.01 (0.46, 2.22)	0.7 (0.36, 1.34)	0.32 (0.1, 1.06)	0.91 (0.39, 2.07)	**0.38 (0.17, 0.84)**	2.37 (0.87, 6.5)	0.71 (0.24, 2.03)	1.25 (0.53, 2.9)	1.81 (0.81, 4.07)	**0.28 (0.14, 0.55)**	**0.24 (0.13, 0.43)**	**0.31 (0.16, 0.6)**	**0.3 (0.19, 0.48)**	1.43 (0.93, 2.17)	0.59 (0.28, 1.21)	3.49 (0.97, 14.06)	**0.45 (0.25, 0.77)**	0.71 (0.24, 2.29)	VTP

Figure S15. League Plot for the NMA of Grade ≥3AEs.

League table presenting ORs with 95% CIs for all pairwise comparisons. Each cell compares the column-defining intervention against the row-defining intervention. An OR with a 95% CI not including 1 indicates a statistically significant difference (P < 0.05). An OR > 1 indicates that the row-defining intervention is favorable, associated with a lower risk of the adverse event. Treatment regimen abbreviations are defined in the List of Abbreviations.

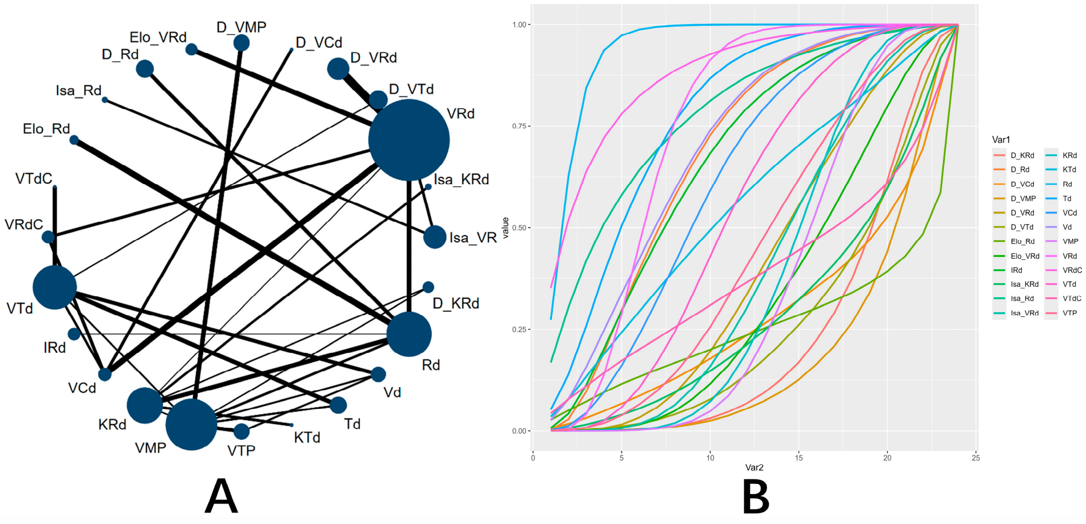

Figure S16. NMA of Treatment Discontinuation Due to Adverse Events.

(A) Network plot of treatment comparisons. The size of each node is proportional to the number of participants assigned to that regimen, and the width of each line (edge) is proportional to the number of trials directly comparing the connected regimens. (B) SUCRA plot. A higher SUCRA value (closer to 100%) indicates that the regimen is associated with a lower risk of treatment discontinuation due to adverse events. Treatment regimen abbreviations are defined in the List of Abbreviations.

D_KRd																							
2.18 (0.84, 5.66)	D_Rd																						
0.93 (0.2, 4.17)	0.43 (0.09, 2)	D_VCd																					
0.88 (0.37, 2.07)	0.4 (0.14, 1.13)	0.94 (0.2, 4.62)	D_VMP																				
1.37 (0.55, 3.34)	0.63 (0.26, 1.49)	1.46 (0.34, 6.76)	1.55 (0.56, 4.3)	D_VRd																			
1.02 (0.37, 2.84)	0.47 (0.15, 1.44)	1.08 (0.23, 5.37)	1.16 (0.41, 3.26)	0.75 (0.25, 2.24)	D_VTd																		
0.65 (0.02, 6.7)	0.3 (0.01, 2.69)	0.69 (0.02, 9.29)	0.75 (0.02, 7.82)	0.48 (0.02, 4.68)	0.64 (0.02, 7.1)	Elo_Rd																	
1.22 (0.48, 3.04)	0.56 (0.22, 1.32)	1.3 (0.29, 6.03)	1.39 (0.49, 3.82)	0.89 (0.41, 1.9)	1.2 (0.38, 3.57)	1.86 (0.19, 67.11)	Elo_VRd																
2.13 (0.79, 5.81)	0.98 (0.43, 2.21)	2.3 (0.47, 11.43)	2.42 (0.81, 7.28)	1.56 (0.6, 4.09)	2.1 (0.64, 6.98)	3.27 (0.34,125.96)	1.74 (0.67, 4.74)	IRd															
1.08 (0.33, 3.32)	0.49 (0.14, 1.67)	1.14 (0.21, 6.65)	1.21 (0.34, 4.38)	0.78 (0.24, 2.5)	1.05 (0.27, 4.11)	1.62 (0.14, 60.35)	0.88 (0.26, 2.89)	0.5 (0.13, 1.82)	Isa_KRd														
2.97 (0.8, 11.2)	1.36 (0.37, 5.1)	3.19 (0.55, 19.42)	3.38 (0.83,13.94)	2.16 (0.64, 7.65)	2.95 (0.66,12.68)	4.67 (0.38, 159.16)	2.41 (0.72, 8.66)	1.37 (0.36, 5.53)	2.75 (0.61, 13.56)	Isa_Rd													
1.37 (0.58, 3.13)	0.62 (0.27, 1.41)	1.46 (0.35, 6.36)	1.55 (0.59, 4.01)	1 (0.5, 1.99)	1.36 (0.46, 3.81)	2.08 (0.22, 66.64)	1.12 (0.56, 2.29)	0.64 (0.25, 1.58)	1.27 (0.41, 4.13)	0.46 (0.16, 1.23)	Isa_VRd												
1.32 (0.7, 2.44)	0.6 (0.28, 1.31)	1.41 (0.35, 6.06)	1.49 (0.66, 3.39)	0.96 (0.48, 1.97)	1.3 (0.49, 3.37)	2.01 (0.21, 65.54)	1.08 (0.53, 2.27)	0.62 (0.26, 1.44)	1.22 (0.48, 3.32)	0.45 (0.13, 1.45)	0.96 (0.52, 1.82)	KRd											
1.73 (0.49, 6.13)	0.79 (0.21, 3.04)	1.86 (0.31,11.55)	1.96 (0.51, 7.9)	1.26 (0.35, 4.68)	1.71 (0.41, 7.43)	2.7 (0.22, 91.48)	1.41 (0.4, 5.49)	0.8 (0.2, 3.33)	1.62 (0.38, 7.29)	0.58 (0.12, 3.04)	1.25 (0.37, 4.56)	1.31 (0.45, 3.96)	KTd										
**4.13 (1.89, 9.24)**	**1.89 (1.15, 3.17)**	**4.4 (1.06, 19.81)**	**4.68 (1.93,11.82)**	**3.02 (1.49, 6.32)**	**4.08 (1.48, 11.24)**	6.26 (0.73,210.33)	**3.37 (1.65, 7.29)**	**1.92 (1.04, 3.67)**	**3.84 (1.27,12.37)**	1.4 (0.41, 4.63)	**3.01 (1.6, 5.93)**	**3.14 (1.76, 5.71)**	2.4 (0.69, 8.07)	Rd									
**2.58 (1.04, 6.39)**	1.18 (0.41, 3.35)	2.75 (0.61, 13.1)	**2.94 (1.17, 7.38)**	1.89 (0.68, 5.26)	**2.54 (1.07, 5.97)**	3.94 (0.38, 149.96)	2.12 (0.76, 6.1)	1.2 (0.39, 3.64)	2.42 (0.68, 8.92)	0.87 (0.21, 3.51)	1.88 (0.72, 5.05)	1.96 (0.83, 4.62)	1.49 (0.38, 5.91)	0.63 (0.25, 1.55)	Td								
1.92 (0.75, 4.71)	0.88 (0.34, 2.31)	2.03 (0.62, 7.31)	2.18 (0.8, 5.88)	1.4 (0.57, 3.39)	1.89 (0.7, 5.02)	2.95 (0.29, 101.23)	1.58 (0.64, 3.91)	0.9 (0.32, 2.51)	1.8 (0.52, 6.3)	0.64 (0.17, 2.39)	1.4 (0.61, 3.19)	1.46 (0.67, 3.14)	1.1 (0.29, 4.22)	0.47 (0.2, 1.04)	0.75 (0.29, 1.86)	VCd							
2.21 (0.92, 5.62)	1.01 (0.35, 2.99)	2.37 (0.51,12.08)	**2.52 (1.04, 6.34)**	1.62 (0.59, 4.7)	2.18 (0.8, 6.1)	3.39 (0.33, 122.86)	1.81 (0.64, 5.5)	1.04 (0.35, 3.2)	2.05 (0.59, 7.85)	0.75 (0.18, 3.21)	1.61 (0.61, 4.56)	1.67 (0.73, 4.11)	1.28 (0.32, 5.19)	0.54 (0.21, 1.38)	0.86 (0.35, 2.23)	1.16 (0.43, 3.23)	Vd						
1.26 (0.69, 2.34)	0.58 (0.25, 1.32)	1.34 (0.33, 5.87)	1.43 (0.79, 2.66)	0.92 (0.41, 2.08)	1.23 (0.53, 2.91)	1.91 (0.2, 64.02)	1.03 (0.45, 2.43)	0.59 (0.24, 1.47)	1.17 (0.39, 3.68)	0.42 (0.12, 1.5)	0.92 (0.44, 1.99)	0.95 (0.55, 1.67)	0.73 (0.21, 2.45)	**0.3 (0.15, 0.59)**	**0.49 (0.24, 0.99)**	0.66 (0.3, 1.45)	0.57 (0.28, 1.09)	VMP					
**2.34 (1.12, 4.84)**	1.07 (0.52, 2.13)	2.48 (0.64, 10.36)	**2.66 (1.12, 6.32)**	**1.71 (1, 2.95)**	2.3 (0.87, 6)	3.55 (0.39, 113)	**1.92 (1.12, 3.38)**	1.1 (0.49, 2.41)	2.18 (0.77, 6.46)	0.79 (0.25, 2.34)	**1.71 (1.11, 2.66)**	**1.78 (1.12, 2.79)**	1.36 (0.41, 4.28)	**0.57 (0.34, 0.91)**	0.9 (0.38, 2.16)	1.22 (0.61, 2.44)	1.06 (0.42, 2.57)	1.86 (1, 3.49)	VRd				
**3.97 (1.09, 15.4)**	1.82 (0.49, 7.21)	4.22 (0.84,23.63)	**4.51 (1.17, 18.49)**	2.9 (0.87, 10.51)	3.91 (0.98,16.44)	6.21 (0.51,244.61)	3.26 (0.98, 12.24)	1.86 (0.48, 7.55)	3.75 (0.82, 17.7)	1.35 (0.27, 6.5)	2.92 (0.9, 10.17)	3.03 (0.94,10.53)	2.29 (0.46, 11.97)	0.96 (0.29, 3.37)	1.53 (0.41, 6.2)	2.04 (0.68, 6.93)	1.79 (0.45, 7.32)	3.17 (0.94,11.35)	1.7 (0.57, 5.52)	VRdC			
1.67 (0.73, 3.77)	0.77 (0.29, 1.98)	1.78 (0.43, 7.75)	1.9 (0.82, 4.34)	1.22 (0.49, 3.05)	1.65 (0.9, 3.02)	2.56 (0.25, 95.18)	1.37 (0.54, 3.55)	0.78 (0.28, 2.15)	1.56 (0.46, 5.37)	0.56 (0.15, 2.12)	1.22 (0.53, 2.91)	1.27 (0.61, 2.67)	0.97 (0.26, 3.55)	**0.4 (0.18, 0.89)**	0.65 (0.35, 1.18)	0.87 (0.41, 1.9)	0.76 (0.33, 1.64)	1.33 (0.74, 2.37)	0.71 (0.34, 1.51)	0.42 (0.11, 1.45)	VTd		
1.22 (0.17, 7.24)	0.56 (0.07, 3.63)	1.27 (0.13, 11.34)	1.36 (0.19, 8.19)	0.89 (0.11, 5.62)	1.18 (0.18, 6.68)	1.88 (0.1, 104.47)	1 (0.13, 6.37)	0.57 (0.07, 3.86)	1.15 (0.12, 8.6)	0.4 (0.04, 3.37)	0.89 (0.12, 5.49)	0.92 (0.13, 5.34)	0.69 (0.07, 5.66)	0.29 (0.04, 1.77)	0.47 (0.07, 2.59)	0.63 (0.09, 3.73)	0.55 (0.07, 3.22)	0.97 (0.14, 5.29)	0.52 (0.07, 3.01)	0.31 (0.03, 2.36)	0.72 (0.12, 3.59)	VTdC	
1.45 (0.65, 3.27)	0.66 (0.25, 1.79)	1.54 (0.34, 7.3)	1.64 (0.72, 3.78)	1.06 (0.41, 2.85)	1.42 (0.54, 3.91)	2.21 (0.22, 80.23)	1.19 (0.44, 3.27)	0.68 (0.24, 1.93)	1.34 (0.4, 4.77)	0.49 (0.12, 1.91)	1.05 (0.43, 2.69)	1.1 (0.52, 2.38)	0.84 (0.22, 3.16)	**0.35 (0.15, 0.81)**	0.56 (0.24, 1.37)	0.76 (0.3, 1.95)	0.65 (0.31, 1.35)	1.15 (0.67, 1.99)	0.62 (0.28, 1.41)	0.36 (0.09, 1.36)	0.86 (0.4, 1.92)	1.18 (0.2, 9.01)	VTP

Figure S17. League Plot for the NMA of Treatment Discontinuation Due to Adverse Events.

League table presenting ORs with 95% CIs for all pairwise comparisons. Each cell compares the column-defining intervention against the row-defining intervention. An OR with a 95% CI not including 1 indicates a statistically significant difference (P < 0.05). An OR > 1 indicates that the row-defining intervention is favorable, associated with a lower risk of the adverse event. Treatment regimen abbreviations are defined in the List of Abbreviations.

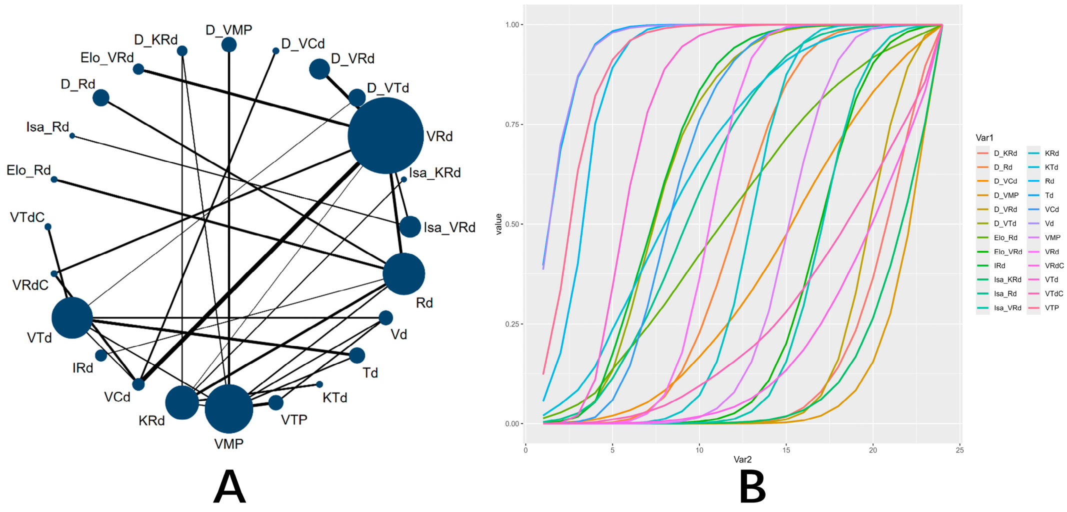

Figure S18. NMA of Hematologic Toxicity.

(A) Network plot of treatment comparisons. The size of each node is proportional to the number of participants assigned to that regimen, and the width of each line (edge) is proportional to the number of trials directly comparing the connected regimens. (B) SUCRA plot. A higher SUCRA value (closer to 100%) indicates that the regimen is associated with a lower risk of hematologic toxicity. Treatment regimen abbreviations are defined in the List of Abbreviations.

D_KRd																							
**2.05 (1.07, 3.89)**	D_Rd																						
1.58 (0.58, 4.35)	0.78 (0.29, 2.09)	D_VCd																					
0.86 (0.49, 1.51)	**0.42 (0.23, 0.79)**	0.55 (0.21, 1.47)	D_VMP																				
1.1 (0.61, 2)	**0.54 (0.32, 0.92)**	0.69 (0.27, 1.82)	1.28 (0.72, 2.28)	D_VRd																			
**2.96 (1.47, 6.04)**	1.44 (0.71, 3.04)	1.87 (0.72, 4.89)	**3.43 (1.82, 6.49)**	**2.67 (1.38, 5.29)**	D_VTd																		
2.19 (0.69, 6.8)	1.08 (0.37, 3)	1.39 (0.35, 5.4)	2.54 (0.8, 7.67)	1.99 (0.67, 5.74)	0.74 (0.22, 2.4)	Elo_Rd																	
1.4 (0.73, 2.63)	0.68 (0.39, 1.21)	0.88 (0.33, 2.38)	1.61 (0.86, 3)	1.26 (0.81, 1.94)	**0.47 (0.23, 0.96)**	0.63 (0.21, 1.96)	Elo_VRd																
**2.99 (1.48, 5.96)**	1.46 (0.89, 2.43)	1.89 (0.67, 5.27)	**3.45 (1.77, 6.69)**	**2.7 (1.51, 4.79)**	1 (0.47, 2.14)	1.35 (0.48, 4)	**2.14 (1.14, 4)**	IRd															
0.91 (0.45, 1.82)	**0.45 (0.22, 0.91)**	0.57 (0.19, 1.68)	1.05 (0.5, 2.16)	0.83 (0.43, 1.56)	**0.31 (0.13, 0.69)**	0.41 (0.13, 1.41)	0.65 (0.33, 1.29)	0.31 (0.14, 0.65)	Isa_KRd														
**2.5 (1.11, 5.65)**	1.22 (0.57, 2.65)	1.58 (0.52, 4.72)	**2.88 (1.3, 6.45)**	**2.26 (1.15, 4.44)**	0.84 (0.35, 2.04)	1.13 (0.34, 3.96)	1.79 (0.89, 3.69)	0.83 (0.37, 1.92)	**2.73 (1.16, 6.58)**	Isa_Rd													
1.38 (0.76, 2.51)	0.67 (0.39, 1.16)	0.87 (0.34, 2.28)	1.59 (0.89, 2.9)	1.25 (0.85, 1.83)	**0.47 (0.23, 0.91)**	0.63 (0.22, 1.9)	0.99 (0.63, 1.57)	**0.46 (0.26, 0.84)**	1.51 (0.79, 2.97)	**0.55 (0.32, 0.95)**	Isa_VRd												
**1.94 (1.19, 3.13)**	0.95 (0.58, 1.58)	1.22 (0.49, 3.14)	**2.24 (1.35, 3.73)**	**1.76 (1.19, 2.62)**	0.66 (0.34, 1.22)	0.88 (0.31, 2.64)	1.39 (0.88, 2.22)	0.65 (0.37, 1.14)	**2.13 (1.29, 3.58)**	0.78 (0.39, 1.51)	1.4 (0.93, 2.12)	KRd											
**2.79 (1.12, 7.11)**	1.37 (0.53, 3.46)	1.76 (0.53, 5.99)	**3.23 (1.27, 8.32)**	**2.53 (1.06, 6.15)**	0.95 (0.34, 2.57)	1.27 (0.35, 4.78)	2.01 (0.82, 4.97)	0.94 (0.36, 2.43)	**3.06 (1.22, 7.92)**	1.12 (0.4, 3.14)	2.03 (0.84, 4.92)	1.44 (0.67, 3.2)	KTd										
**4.71 (2.69, 8.34)**	**2.31 (1.72, 3.15)**	**2.97 (1.16, 7.77)**	**5.46 (3.19, 9.36)**	**4.28 (2.8, 6.49)**	1.6 (0.82, 3.04)	2.14 (0.81, 5.92)	**3.38 (2.1, 5.5)**	**1.58 (1.06, 2.36)**	**5.16 (2.73, 10)**	1.89 (0.93, 3.81)	**3.42 (2.2, 5.32)**	**2.44 (1.64, 3.61)**	1.68 (0.7, 4.09)	Rd									
**6.21 (3.18, 12.41)**	**3.04 (1.53, 6.17)**	**3.93 (1.54, 10.27)**	**7.25 (4.02, 13.1)**	**5.64 (2.95, 10.86)**	**2.1 (1.23, 3.59)**	2.85 (0.9, 9.72)	**4.46 (2.24, 9.07)**	**2.09 (1, 4.44)**	**6.82 (3.14, 15.38)**	**2.5 (1.04, 5.96)**	**4.52 (2.34, 8.81)**	**3.21 (1.76, 5.93)**	2.24 (0.82, 5.97)	1.32 (0.71, 2.49)	Td								
**2.72 (1.42, 5.28)**	1.33 (0.71, 2.54)	1.71 (0.8, 3.67)	**3.15 (1.71, 5.8)**	**2.47 (1.4, 4.31)**	0.92 (0.52, 1.62)	1.24 (0.4, 3.99)	**1.95 (1.07, 3.65)**	0.92 (0.46, 1.82)	**2.98 (1.42, 6.38)**	1.09 (0.49, 2.4)	**1.97 (1.12, 3.47)**	1.41 (0.81, 2.42)	0.98 (0.37, 2.52)	0.58 (0.33, 1.01)	**0.44 (0.24, 0.77)**	VCd							
**6.24 (3.18, 12.42)**	**3.05 (1.51, 6.21)**	**3.93 (1.44, 10.76)**	**7.23 (4.04, 13.03)**	**5.67 (2.88, 10.99)**	**2.1 (1.1, 4.01)**	2.85 (0.87, 9.53)	**4.47 (2.19, 9.28)**	**2.1 (1, 4.42)**	**6.83 (3.11, 15.32)**	**2.52 (1.04, 5.98)**	**4.54 (2.26, 8.94)**	3.22 (1.72, 6.03)	2.24 (0.82, 6)	1.32 (0.7, 2.53)	1 (0.54, 1.88)	**2.28 (1.2, 4.5)**	Vd						
**1.62 (1.01, 2.6)**	0.8 (0.47, 1.35)	1.03 (0.41, 2.6)	**1.88 (1.37, 2.58)**	1.47 (0.9, 2.39)	**0.55 (0.31, 0.95)**	0.74 (0.26, 2.25)	1.17 (0.68, 2.03)	0.55 (0.3, 0.98)	1.78 (0.94, 3.45)	0.65 (0.31, 1.35)	1.18 (0.71, 1.95)	0.84 (0.56, 1.24)	0.58 (0.24, 1.39)	**0.34 (0.22, 0.53)**	**0.26 (0.16, 0.43)**	0.6 (0.36, 1.01)	**0.26 (0.16, 0.43)**	VMP					
**2.28 (1.34, 3.87)**	1.11 (0.71, 1.76)	1.44 (0.58, 3.62)	**2.64 (1.57, 4.42)**	**2.06 (1.59, 2.66)**	0.77 (0.41, 1.42)	1.03 (0.37, 3.02)	**1.64 (1.15, 2.34)**	0.76 (0.45, 1.28)	**2.5 (1.4, 4.56)**	0.91 (0.49, 1.69)	**1.65 (1.24, 2.21)**	1.18 (0.87, 1.57)	0.81 (0.35, 1.86)	**0.48 (0.35, 0.67)**	**0.37 (0.2, 0.66)**	0.84 (0.51, 1.36)	**0.37 (0.2, 0.68)**	1.4 (0.93, 2.12)	VRd				
1.07 (0.41, 2.79)	0.53 (0.21, 1.35)	0.68 (0.22, 2.06)	1.24 (0.48, 3.13)	0.97 (0.41, 2.27)	0.36 (0.14, 0.92)	0.48 (0.13, 1.83)	0.77 (0.31, 1.87)	0.36 (0.14, 0.95)	1.17 (0.43, 3.19)	0.43 (0.15, 1.2)	0.78 (0.33, 1.85)	0.55 (0.23, 1.3)	0.38 (0.12, 1.24)	**0.23 (0.09, 0.55)**	**0.17 (0.07, 0.44)**	**0.4 (0.17, 0.89)**	**0.17 (0.07, 0.46)**	0.66 (0.27, 1.58)	0.47 (0.21, 1.06)	VRdC			
**3.44 (1.88, 6.39)**	1.69 (0.9, 3.22)	2.18 (0.9, 5.29)	**4.01 (2.36, 6.81)**	**3.12 (1.77, 5.55)**	1.16 (0.82, 1.64)	1.57 (0.51, 5.07)	**2.47 (1.34, 4.65)**	1.16 (0.59, 2.3)	**3.78 (1.84, 8.04)**	1.38 (0.62, 3.09)	**2.5 (1.41, 4.52)**	**1.77 (1.06, 3.03)**	1.23 (0.48, 3.19)	0.73 (0.43, 1.28)	**0.55 (0.37, 0.82)**	1.26 (0.81, 2.02)	**0.55 (0.32, 0.96)**	**2.13 (1.39, 3.24)**	1.51 (0.91, 2.55)	**3.24 (1.34, 7.79)**	VTd		
1.26 (0.43, 3.68)	0.62 (0.21, 1.79)	0.79 (0.23, 2.79)	1.45 (0.52, 4.01)	1.14 (0.4, 3.17)	0.43 (0.16, 1.07)	0.58 (0.14, 2.45)	0.91 (0.31, 2.6)	0.42 (0.14, 1.25)	1.38 (0.44, 4.35)	0.51 (0.15, 1.63)	0.91 (0.32, 2.56)	0.65 (0.23, 1.78)	0.45 (0.13, 1.61)	**0.27 (0.09, 0.74)**	**0.2 (0.07, 0.52)**	0.46 (0.17, 1.24)	**0.2 (0.07, 0.57)**	0.78 (0.29, 1.99)	0.55 (0.2, 1.51)	1.19 (0.33, 3.94)	0.37 (0.15, 0.86)	VTdC	
**5.27 (2.76, 10.11)**	**2.58 (1.32, 5.14)**	**3.34 (1.21, 9.17)**	**6.08 (3.63, 10.46)**	**4.76 (2.52, 9.21)**	1.78 (0.9, 3.5)	2.4 (0.77, 7.91)	**3.77 (1.93, 7.64)**	1.77 (0.86, 3.62)	**5.76 (2.68, 12.57)**	2.11 (0.9, 4.94)	**3.81 (1.98, 7.45)**	**2.72 (1.53, 4.89)**	1.89 (0.71, 4.97)	1.12 (0.61, 2.06)	0.84 (0.44, 1.6)	**1.93 (1.01, 3.77)**	0.84 (0.48, 1.5)	**3.24 (2.14, 5.04)**	**2.31 (1.3, 4.25)**	**4.95 (1.87, 12.91)**	1.53 (0.86, 2.73)	**4.19 (1.47, 12.06)**	VTP

Figure S19. League Plot for the NMA of Hematologic Toxicity.

League table presenting ORs with 95% CIs for all pairwise comparisons. Each cell compares the column-defining intervention against the row-defining intervention. An OR with a 95% CI not including 1 indicates a statistically significant difference ($P < 0.05$). An OR > 1 indicates that the row-defining intervention is favorable, associated with a lower risk of the adverse event. Treatment regimen abbreviations are defined in the List of Abbreviations.

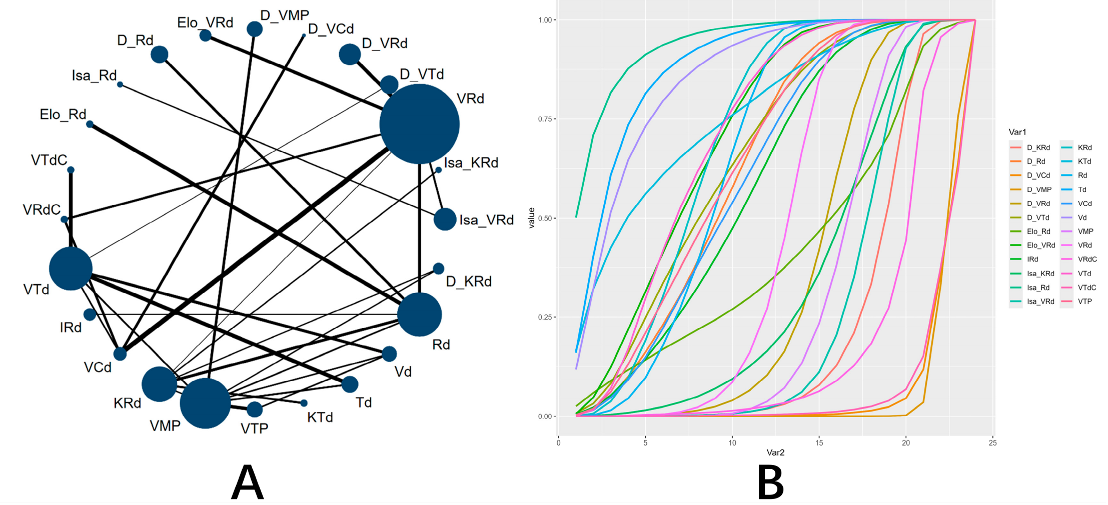

Figure S20. NMA of Infection Risk.

(A) Network plot of treatment comparisons. The size of each node is proportional to the number of participants assigned to that regimen, and the width of each line (edge) is proportional to the number of trials directly comparing the connected regimens. (B) SUCRA plot. A higher SUCRA value (closer to 100%) indicates that the regimen is associated with a lower risk of infection. Treatment regimen abbreviations are defined in the List of Abbreviations.

D_KRd																							
2.09 (0.99, 4.36)	D_Rd																						
0.33 (0.08, 1.14)	**0.16 (0.04, 0.55)**	D_VCd																					
**0.35 (0.19, 0.65)**	**0.17 (0.08, 0.33)**	1.06 (0.32, 4.05)	D_VMP																				
1.42 (0.74, 2.74)	0.68 (0.37, 1.23)	**4.33 (1.34, 15.93)**	**4.08 (2.12, 7.78)**	D_VRd																			
2.2 (0.99, 4.76)	1.05 (0.46, 2.37)	**6.69 (2.01, 24.97)**	**6.28 (3.05, 12.97)**	1.54 (0.72, 3.3)	D_VTd																		
1.4 (0.33, 5.4)	0.68 (0.18, 2.32)	4.3 (0.75, 24.28)	4.03 (0.99, 15.31)	0.99 (0.25, 3.57)	0.64 (0.15, 2.65)	Elo_Rd																	
**2.34 (1.14, 4.76)**	1.12 (0.56, 2.17)	**7.17 (2.11, 27.6)**	**6.69 (3.33, 13.63)**	**1.64 (1.02, 2.71)**	1.07 (0.47, 2.42)	1.67 (0.45, 6.86)	Elo_VRd																
1.98 (0.89, 4.3)	0.94 (0.52, 1.7)	**6.01 (1.68, 23.6)**	**5.65 (2.63, 12.05)**	1.39 (0.71, 2.68)	0.9 (0.37, 2.15)	1.39 (0.39, 5.56)	0.84 (0.41, 1.73)	IRd															
1.32 (0.6, 2.91)	0.63 (0.29, 1.44)	**4.06 (1.11, 16.46)**	**3.81 (1.68, 8.46)**	0.93 (0.46, 1.9)	0.61 (0.25, 1.51)	0.94 (0.24, 4.05)	0.56 (0.26, 1.23)	0.67 (0.29, 1.59)	Isa_KRd														
**4.49 (1.77, 11.59)**	2.14 (0.88, 5.38)	**13.77 (3.58, 58.34)**	**12.84 (5.11, 33.32)**	**3.16 (1.48, 7)**	2.06 (0.76, 5.71)	3.2 (0.75, 14.78)	1.91 (0.84, 4.51)	2.28 (0.89, 5.9)	**3.39 (1.28, 9.01)**	Isa_Rd													
1.16 (0.58, 2.3)	0.55 (0.3, 1.04)	**3.51 (1.08, 12.87)**	**3.33 (1.67, 6.55)**	0.82 (0.52, 1.28)	0.53 (0.24, 1.17)	0.83 (0.23, 3.28)	**0.49 (0.29, 0.86)**	0.59 (0.3, 1.18)	0.87 (0.42, 1.82)	**0.26 (0.13, 0.48)**	Isa_VRd												
**2.26 (1.3, 3.99)**	1.09 (0.61, 1.93)	**6.87 (2.14, 25.86)**	**6.52 (3.66, 11.49)**	**1.6 (1.04, 2.47)**	1.04 (0.5, 2.13)	1.62 (0.45, 6.27)	0.97 (0.57, 1.62)	1.15 (0.61, 2.21)	1.72 (0.97, 2.98)	0.51 (0.22, 1.11)	**1.96 (1.2, 3.14)**	KRd											
2.98 (0.94, 9.32)	1.42 (0.45, 4.51)	**9.12 (1.98, 46.12)**	**8.58 (2.68, 26.88)**	2.1 (0.69, 6.15)	1.36 (0.4, 4.65)	2.12 (0.42, 11.46)	1.28 (0.4, 3.94)	1.51 (0.45, 4.97)	2.24 (0.71, 6.91)	0.67 (0.18, 2.32)	2.58 (0.83, 7.64)	1.31 (0.48, 3.51)	KTd										
**2.12 (1.12, 3.99)**	1.01 (0.71, 1.46)	**6.46 (2, 23.22)**	**6.09 (3.35, 11.09)**	1.49 (0.95, 2.4)	0.97 (0.47, 2.05)	1.51 (0.46, 5.52)	0.9 (0.53, 1.58)	1.08 (0.68, 1.73)	1.6 (0.77, 3.25)	0.47 (0.21, 1.06)	**1.84 (1.1, 3.04)**	0.94 (0.6, 1.46)	0.72 (0.24, 2.17)	Rd									
**3.35 (1.54, 7.46)**	1.6 (0.71, 3.72)	**10.24 (3.02, 38.99)**	**9.61 (4.86, 19.79)**	**2.35 (1.1, 5.19)**	1.53 (0.8, 2.99)	2.42 (0.6, 10.41)	1.42 (0.63, 3.36)	1.69 (0.72, 4.15)	**2.54 (1.02, 6.29)**	0.74 (0.27, 2.05)	**2.88 (1.32, 6.48)**	1.48 (0.73, 3.11)	1.13 (0.34, 3.85)	1.57 (0.76, 3.36)	Td								
2.02 (0.95, 4.28)	0.97 (0.45, 2.03)	**6.09 (2.29, 19.34)**	**5.82 (2.84, 11.81)**	1.42 (0.74, 2.71)	0.92 (0.47, 1.81)	1.45 (0.37, 5.94)	0.86 (0.42, 1.74)	1.02 (0.46, 2.3)	1.53 (0.65, 3.52)	0.45 (0.17, 1.13)	1.73 (0.87, 3.43)	0.89 (0.47, 1.66)	0.68 (0.21, 2.21)	0.95 (0.49, 1.81)	0.6 (0.29, 1.24)	VCd							
**3.12 (1.45, 6.83)**	1.49 (0.65, 3.39)	**9.46 (2.7, 38.63)**	**8.93 (4.63, 17.27)**	**2.19 (1, 4.87)**	1.42 (0.66, 3.13)	2.23 (0.55, 9.36)	1.33 (0.58, 3.13)	1.58 (0.66, 3.87)	2.35 (0.94, 5.85)	0.69 (0.25, 1.91)	**2.69 (1.21, 6.07)**	1.37 (0.67, 2.83)	1.05 (0.31, 3.55)	1.47 (0.7, 3.05)	0.93 (0.43, 2.01)	1.55 (0.7, 3.41)	Vd						
1.27 (0.75, 2.14)	0.61 (0.33, 1.11)	**3.87 (1.22, 14.09)**	**3.67 (2.58, 5.14)**	0.9 (0.52, 1.57)	0.58 (0.31, 1.09)	0.91 (0.25, 3.5)	0.55 (0.29, 1.01)	0.65 (0.33, 1.28)	0.96 (0.46, 1.97)	**0.28 (0.12, 0.67)**	1.1 (0.61, 1.97)	**0.56 (0.35, 0.89)**	0.43 (0.14, 1.3)	**0.6 (0.37, 0.97)**	**0.38 (0.2, 0.69)**	0.63 (0.34, 1.17)	**0.41 (0.23, 0.71)**	VMP					
1.65 (0.9, 2.97)	0.79 (0.47, 1.34)	**4.99 (1.6, 17.88)**	**4.73 (2.62, 8.45)**	1.16 (0.88, 1.54)	0.75 (0.37, 1.53)	1.18 (0.34, 4.51)	0.7 (0.47, 1.05)	0.84 (0.46, 1.53)	1.25 (0.64, 2.36)	**0.37 (0.17, 0.75)**	**1.42 (1, 2.01)**	0.73 (0.52, 1.01)	0.55 (0.2, 1.61)	0.78 (0.53, 1.12)	**0.49 (0.24, 0.99)**	0.82 (0.45, 1.47)	0.53 (0.25, 1.09)	1.29 (0.81, 2.08)	VRd				
0.78 (0.29, 2.1)	**0.38 (0.14, 0.99)**	2.38 (0.63, 9.62)	2.26 (0.82, 6)	0.55 (0.23, 1.32)	**0.36 (0.12, 0.97)**	0.56 (0.12, 2.68)	**0.34 (0.13, 0.82)**	0.4 (0.14, 1.1)	0.6 (0.21, 1.65)	**0.17 (0.05, 0.52)**	0.67 (0.27, 1.66)	**0.35 (0.14, 0.82)**	**0.26 (0.07, 1)**	**0.37 (0.15, 0.9)**	**0.23 (0.08, 0.64)**	**0.39 (0.16, 0.91)**	**0.25 (0.08, 0.73)**	0.62 (0.24, 1.55)	0.48 (0.2, 1.08)	VRdC			
**2.34 (1.17, 4.61)**	1.12 (0.53, 2.33)	**7.12 (2.28, 25.32)**	**6.69 (3.66, 12.24)**	1.64 (0.85, 3.21)	1.07 (0.73, 1.56)	1.66 (0.43, 6.87)	0.99 (0.49, 2.07)	1.18 (0.55, 2.66)	1.76 (0.77, 3.96)	0.52 (0.2, 1.3)	**2.02 (1.01, 3.98)**	1.03 (0.56, 1.9)	0.79 (0.24, 2.52)	1.1 (0.58, 2.08)	0.69 (0.4, 1.18)	1.16 (0.66, 2.03)	0.75 (0.37, 1.44)	**1.82 (1.13, 2.99)**	1.42 (0.78, 2.59)	**2.97 (1.18, 7.93)**	VTd		
0.33 (0.07, 1.25)	**0.16 (0.03, 0.61)**	0.99 (0.17, 5.58)	0.95 (0.22, 3.42)	**0.23 (0.05, 0.88)**	**0.15 (0.04, 0.51)**	0.23 (0.04, 1.42)	**0.14 (0.03, 0.53)**	**0.17 (0.03, 0.67)**	0.25 (0.05, 1.01)	**0.07 (0.01, 0.32)**	0.28 (0.06, 1.08)	**0.15 (0.03, 0.53)**	**0.11 (0.02, 0.57)**	**0.15 (0.03, 0.57)**	**0.1 (0.02, 0.36)**	**0.16 (0.04, 0.6)**	**0.1 (0.02, 0.39)**	**0.26 (0.06, 0.89)**	**0.2 (0.04, 0.73)**	0.42 (0.08, 1.91)	**0.14 (0.04, 0.45)**	VTdC	
**2.13 (1.07, 4.2)**	1.02 (0.48, 2.12)	**6.51 (1.91, 25.02)**	**6.11 (3.52, 10.6)**	1.49 (0.75, 3.04)	0.97 (0.47, 2.07)	1.52 (0.39, 6.36)	0.91 (0.43, 1.92)	1.08 (0.49, 2.4)	1.6 (0.69, 3.77)	0.48 (0.18, 1.26)	1.83 (0.89, 3.8)	0.94 (0.51, 1.77)	0.71 (0.23, 2.39)	1 (0.52, 1.91)	0.63 (0.3, 1.33)	1.06 (0.51, 2.21)	0.68 (0.37, 1.26)	**1.67 (1.1, 2.59)**	1.29 (0.68, 2.45)	2.71 (0.99, 7.69)	0.91 (0.49, 1.73)	**6.52 (1.76, 29.19)**	VTP

Figure S21. League Plot for the NMA of Infection Risk.

League table presenting ORs with 95% CIs for all pairwise comparisons. Each cell compares the column-defining intervention against the row-defining intervention. An OR with a 95% CI not including 1 indicates a statistically significant difference (P < 0.05). An OR > 1 indicates that the row-defining intervention is favorable, associated with a lower risk of the adverse event. Treatment regimen abbreviations are defined in the List of Abbreviations.

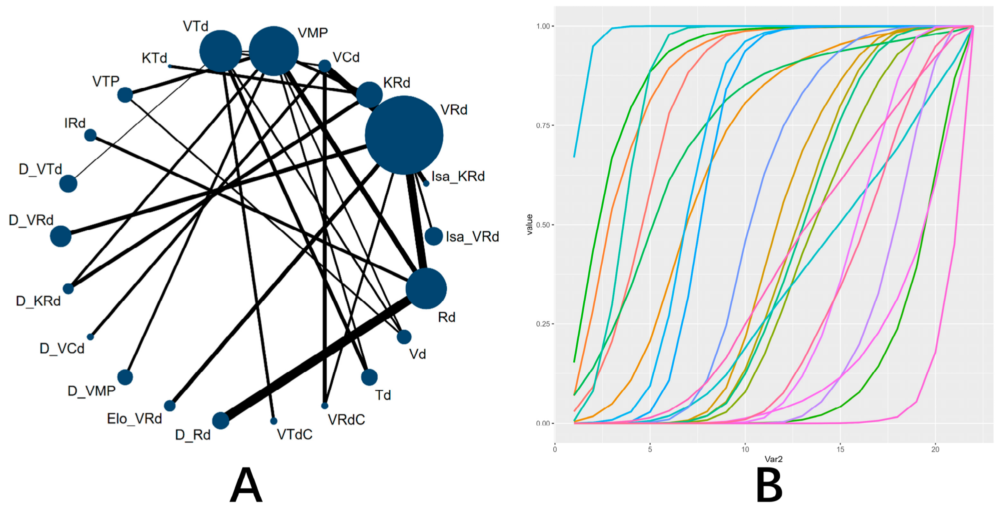

Figure S22. NMA of Neurotoxicity.

(A) Network plot of treatment comparisons. The size of each node is proportional to the number of participants assigned to that regimen, and the width of each line (edge) is proportional to the number of trials directly comparing the connected regimens. (B) SUCRA plot. A higher SUCRA value (closer to 100%) indicates that the regimen is associated with a lower risk of neurotoxicity. Treatment regimen abbreviations are defined in the List of Abbreviations.

D_KRd																					
1.7 (0.23, 11.54)	D_Rd																				
0.5 (0.08, 2.98)	0.29 (0.04, 2.45)	D_VCd																			
**0.23 (0.07, 0.7)**	**0.13 (0.02, 0.76)**	0.45 (0.1, 2.03)	D_VMP																		
**0.2 (0.05, 0.75)**	**0.12 (0.03, 0.58)**	0.4 (0.09, 1.78)	0.87 (0.33, 2.41)	D_VRd																	
**0.19 (0.05, 0.65)**	**0.11 (0.02, 0.64)**	0.37 (0.08, 1.64)	0.81 (0.37, 1.77)	0.93 (0.32, 2.53)	D_VTd																
**0.09 (0.02, 0.37)**	**0.05 (0.01, 0.27)**	**0.18 (0.04, 0.84)**	0.4 (0.14, 1.16)	**0.45 (0.26, 0.81)**	0.49 (0.17, 1.49)	Elo_VRd															
2.21 (0.3, 16.27)	1.28 (0.22, 8.04)	4.38 (0.55, 36.41)	**9.59 (1.74, 56.87)**	**11.02 (2.14, 56.11)**	**11.86 (2.1, 73.07)**	**24.38 (4.6, 132.02)**	IRd														
0.78 (0.06, 7.48)	0.45 (0.03, 5.62)	1.54 (0.12, 17.28)	3.41 (0.33, 28.61)	3.84 (0.41, 30.37)	4.19 (0.39, 37.07)	8.41 (0.89, 68.97)	0.35 (0.02, 4.35)	Isa_KRd													
**0.2 (0.05, 0.75)**	**0.11 (0.02, 0.56)**	0.38 (0.09, 1.75)	0.85 (0.32, 2.39)	0.97 (0.6, 1.6)	1.05 (0.38, 3.11)	**2.14 (1.19, 3.9)**	**0.09 (0.02, 0.45)**	0.25 (0.03, 2.35)	Isa_VRd												
1.32 (0.35, 4.86)	0.77 (0.16, 3.98)	2.62 (0.59, 12.39)	**5.67 (2.36, 15.58)**	**6.55 (3.56, 12.42)**	**7.08 (2.7, 20.41)**	**14.49 (7.26, 29.8)**	0.6 (0.11, 3.08)	1.71 (0.24, 14.63)	**6.76 (3.59, 12.94)**	KRd											
**0.16 (0.02, 0.92)**	**0.09 (0.01, 0.69)**	0.32 (0.04, 2.07)	0.7 (0.13, 3.1)	0.81 (0.17, 2.84)	0.87 (0.16, 3.93)	1.78 (0.37, 6.61)	**0.07 (0.01, 0.52)**	0.21 (0.02, 2.33)	0.84 (0.18, 3.02)	**0.12 (0.03, 0.37)**	KTd										
4.05 (0.9, 18.16)	2.32 (0.75, 9.17)	**8.05 (1.65, 45.09)**	**17.42 (6.05, 60.3)**	**20.08 (8.3, 54.46)**	**21.65 (6.91, 77.74)**	**44.29 (17.36, 129.53)**	1.83 (0.51, 7.2)	5.29 (0.6, 58.98)	**20.62 (8.44, 56.35)**	**3.05 (1.18, 9.08)**	**25.07 (5.95, 144.79)**	Rd									
0.52 (0.14, 1.78)	0.3 (0.05, 1.76)	1.03 (0.22, 4.51)	**2.25 (1.07, 4.88)**	2.6 (0.89, 7.02)	**2.79 (1.4, 5.75)**	**5.69 (1.88, 16.44)**	0.24 (0.04, 1.32)	0.66 (0.08, 7.3)	2.68 (0.9, 7.25)	0.4 (0.14, 1.02)	3.2 (0.73, 17.26)	**0.13 (0.04, 0.39)**	Td								
0.45 (0.12, 1.64)	0.26 (0.05, 1.44)	0.89 (0.26, 3.11)	1.95 (0.82, 4.89)	2.24 (0.97, 5.13)	**2.42 (1.06, 5.83)**	**4.91 (2.05, 12.37)**	0.2 (0.04, 1.12)	0.58 (0.07, 5.72)	**2.29 (1, 5.35)**	**0.34 (0.14, 0.8)**	**2.79 (0.68, 14.04)**	**0.11 (0.04, 0.32)**	0.86 (0.37, 2.13)	VCd							
**0.27 (0.07, 0.93)**	**0.16 (0.03, 0.92)**	0.54 (0.12, 2.54)	1.18 (0.59, 2.46)	1.36 (0.46, 3.78)	1.46 (0.68, 3.21)	2.98 (0.97, 8.86)	**0.12 (0.02, 0.7)**	0.35 (0.04, 3.61)	1.4 (0.47, 3.91)	**0.21 (0.07, 0.53)**	1.68 (0.37, 9.09)	**0.07 (0.02, 0.21)**	0.53 (0.24, 1.14)	0.61 (0.24, 1.47)	Vd						
**0.12 (0.04, 0.34)**	**0.07 (0.01, 0.37)**	0.24 (0.05, 1.01)	**0.52 (0.36, 0.76)**	0.6 (0.23, 1.44)	0.64 (0.32, 1.27)	1.3 (0.48, 3.38)	**0.05 (0.01, 0.28)**	0.15 (0.02, 1.52)	0.61 (0.23, 1.51)	**0.09 (0.04, 0.2)**	0.74 (0.18, 3.67)	**0.03 (0.01, 0.08)**	**0.23 (0.12, 0.44)**	**0.27 (0.12, 0.58)**	**0.44 (0.24, 0.79)**	VMP					
**0.15 (0.04, 0.55)**	**0.09 (0.02, 0.42)**	0.3 (0.07, 1.3)	0.65 (0.27, 1.74)	0.75 (0.55, 1.05)	0.81 (0.31, 2.27)	**1.66 (1.03, 2.68)**	**0.07 (0.01, 0.33)**	0.19 (0.03, 1.81)	0.77 (0.54, 1.11)	**0.11 (0.07, 0.2)**	0.92 (0.27, 4.16)	**0.04 (0.01, 0.08)**	**0.29 (0.11, 0.81)**	**0.34 (0.16, 0.72)**	0.55 (0.21, 1.55)	1.26 (0.56, 3.08)	VRd				
**0.09 (0.02, 0.42)**	**0.05 (0.01, 0.32)**	**0.18 (0.04, 0.94)**	0.4 (0.12, 1.42)	0.46 (0.17, 1.24)	0.5 (0.14, 1.73)	1 (0.36, 2.89)	**0.04 (0.01, 0.25)**	0.12 (0.01, 1.26)	0.47 (0.18, 1.28)	**0.07 (0.02, 0.2)**	0.57 (0.12, 3.34)	**0.02 (0.01, 0.08)**	**0.18 (0.05, 0.63)**	**0.21 (0.07, 0.58)**	0.34 (0.1, 1.22)	0.78 (0.24, 2.56)	0.61 (0.24, 1.55)	VRdC			
**0.06 (0.02, 0.2)**	**0.04 (0.01, 0.21)**	**0.13 (0.03, 0.52)**	**0.28 (0.15, 0.52)**	**0.32 (0.12, 0.77)**	**0.34 (0.21, 0.54)**	0.7 (0.26, 1.83)	**0.03 (0, 0.15)**	**0.08 (0.01, 0.83)**	**0.33 (0.12, 0.8)**	**0.05 (0.02, 0.11)**	0.39 (0.09, 1.99)	**0.02 (0, 0.04)**	**0.12 (0.07, 0.2)**	**0.14 (0.07, 0.28)**	**0.23 (0.12, 0.43)**	**0.54 (0.32, 0.88)**	**0.42 (0.17, 0.96)**	0.69 (0.21, 2.21)	VTd		
0.2 (0.03, 1.12)	**0.11 (0.01, 0.97)**	0.39 (0.06, 2.57)	0.85 (0.23, 3.42)	0.97 (0.21, 4.68)	1.05 (0.3, 4.16)	2.13 (0.46, 10.78)	**0.09 (0.01, 0.75)**	0.25 (0.02, 3.57)	1 (0.21, 4.8)	**0.15 (0.03, 0.69)**	1.23 (0.19, 9.79)	**0.05 (0.01, 0.27)**	0.38 (0.11, 1.52)	0.43 (0.11, 1.87)	0.73 (0.19, 2.92)	1.64 (0.46, 6.24)	1.29 (0.29, 6.01)	2.11 (0.4, 12)	3.05 (0.96, 10.99)	VTdC	
**0.14 (0.04, 0.46)**	**0.08 (0.01, 0.49)**	0.28 (0.06, 1.29)	0.62 (0.33, 1.17)	0.71 (0.25, 1.95)	0.77 (0.34, 1.75)	1.56 (0.52, 4.54)	**0.06 (0.01, 0.36)**	0.18 (0.02, 1.89)	0.73 (0.25, 2.02)	**0.11 (0.04, 0.28)**	0.88 (0.19, 4.64)	**0.04 (0.01, 0.11)**	**0.27 (0.12, 0.61)**	**0.32 (0.12, 0.77)**	0.52 (0.26, 1.03)	1.2 (0.72, 1.98)	0.94 (0.34, 2.44)	1.54 (0.42, 5.41)	**2.23 (1.12, 4.44)**	0.73 (0.17, 2.77)	VTP

Figure S23. League Plot for the NMA of Neurotoxicity.

League table presenting ORs with 95% CIs for all pairwise comparisons. Each cell compares the column-defining intervention against the row-defining intervention. An OR with a 95% CI not including 1 indicates a statistically significant difference (P < 0.05). An OR > 1 indicates that the row-defining intervention is favorable, associated with a lower risk of the adverse event. Treatment regimen abbreviations are defined in the List of Abbreviations.

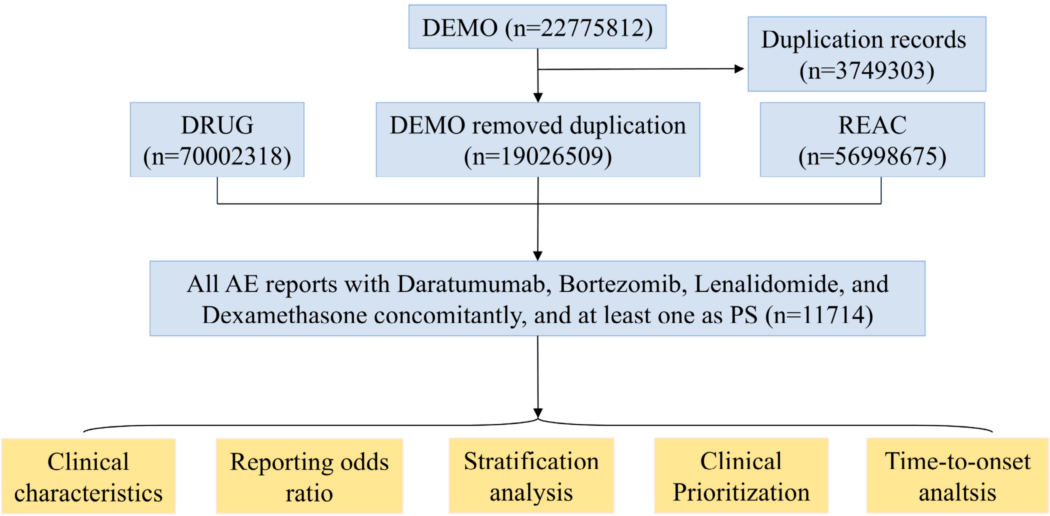

Figure S24. FAERS Database Screening Flowchart.

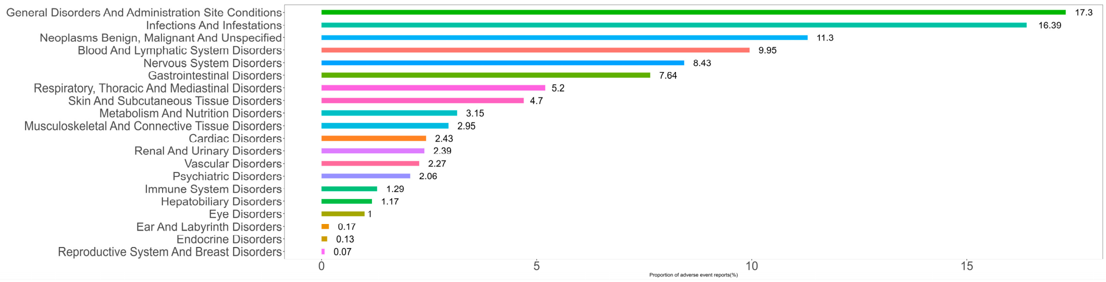

Figure S25. Distribution of Adverse Events by SOC.
